# By-Products of Fruit and Vegetables: Antioxidant Properties of Extractable and Non-Extractable Phenolic Compounds

**DOI:** 10.3390/antiox12020418

**Published:** 2023-02-08

**Authors:** Yu Zeng, Wenyi Zhou, Jiahao Yu, Lei Zhao, Kai Wang, Zhuoyan Hu, Xuwei Liu

**Affiliations:** 1 Guangdong Provincial Key Laboratory of Food Quality and Safety, College of Food Science, South China Agricultural University, Guangzhou 510642, China; 2 School of Food Science and Technology, Zhejiang University of Technology, Hangzhou 310058, China

**Keywords:** wastes, polyphenols, macromolecular antioxidants, structure–activity relationships, natural utilization

## Abstract

Non-extractable phenolic compounds (NEPs), or bound phenolic compounds, represent a crucial component of polyphenols. They are an essential fraction that remains in the residual matrix after the extraction of extractable phenolic compounds (EPs), making them a valuable resource for numerous applications. These compounds encompass a diverse range of phenolic compounds, ranging from low molecular weight phenolic to high polymeric polyphenols attached to other macro molecules, e.g., cell walls and proteins. Their status as natural, green antioxidants have been well established, with numerous studies showcasing their anti-inflammatory, anti-aging, anti-cancer, and hypoglycemic activities. These properties make them a highly desirable alternative to synthetic antioxidants. Fruit and vegetable (F&Veg) wastes, e.g., peels, pomace, and seeds, generated during the harvest, transport, and processing of F&Vegs, are abundant in NEPs and EPs. This review delves into the various types, contents, structures, and antioxidant activities of NEPs and EPs in F&Veg wastes. The relationship between the structure of these compounds and their antioxidant activity is explored in detail, highlighting the importance of structure-activity relationships in the field of natural antioxidants. Their potential applications ranging from functional food and beverage products to nutraceutical and cosmetic products. A glimpse into their bright future as a valuable resource for a greener, healthier, and more sustainable future, and calling for researchers, industrialists, and policymakers to explore their full potential, are elaborated.

## 1. Introduction

In the last decades, there has been a growing interest in polyphenols, mainly because of their potential impact on human health, such as their powerful antioxidant, anti-inflammatory, anti-diabetic, anti-microbial, and other biological activities [1,2,3,4,5]. An important advantage of phenolic compounds over other antioxidants is that they are naturally synthesized by plants and are widely found in a variety of plant foods, e.g., F&Vegs [6]. In contrast, F&Vegs are an essential part of an individual’s daily diet and are consumed in large quantities on a daily basis, making phenolic compounds one of the most abundant antioxidants in the human diet, with a total daily intake that might be as high as 1 g. Phenolic compounds are also known to be more important natural antioxidants than L-ascorbic acid and carotenoids in terms of antioxidant capacity [5,7,8,9]. Phenolic compounds play an important role in the prevention of several chronic diseases related to oxidative stress, e.g., cancer, cardiovascular diseases, and neurodegenerative diseases [10,11]. Since most widely used synthetic antioxidants, such as butylated hydroxyanisole (BHA) and butylated hydroxytoluene (BHT), are suspected to degrade during industrial production to produce harmful or even carcinogenic substances that threaten human health and safety [12]. Therefore, the application of natural antioxidants similar to polyphenols might be a suitable alternative to replace synthetic antioxidants, not only because of their safety but also considering the protection of the added substance from the harmful effects of natural oxidation [13].

Plant polyphenols are a family of antioxidants composed of different subclasses (phenolic acids, flavonoids, stilbene, lignans, etc.) [14,15]. However, the total phenolic content determined by chemical methods is often lower than its true value because the polyphenols found and determined in the extract correspond to only a small fraction of all phenolic substances, i.e., extractable phenolic compounds (EPs). The residue typically left after EP extraction contains neglected polyphenols, the so-called non-extractable phenolics (NEPs). It contains two main groups: (1) large polymeric polyphenols and (2) individual polyphenols linked to macromolecules, e.g., proteins and polysaccharides via hydrophobic interactions as well as hydrogen and/or covalent bonds. Although only a minority of the available literature on F&Veg polyphenols addresses NEPs, the available data indicate that in many F&Vegs, the content of NEPs even exceeds that of EPs [16,17]. NEPs accounted for more than 50% of the total polyphenol content in nearly half of the ten common fruit peels tested, and high-performance liquid chromatography (HPLC) analysis showed that NEPs consisted mainly of phenolic acids, flavanols, and flavonols [18].

F&Vegs are an important part of the human diet and have always been a rich source of polyphenols. The rapid growth of the world’s population, the rapid expansion of cities, changes in eating habits, and many other factors have contributed to this important food group becoming one of the most consumed commodities in the world [14,19,20]. Meanwhile, the loss and waste of F&Vegs are staggering. Global horticultural products are wasted up to 500 million tons per year, accounting for approximately 60% of world food waste [14]. Because fresh F&Vegs are a highly hydrated, perishable, and fragile product, each step of the supply chain, from harvesting, grading, processing, and transportation to disposal in the home kitchen, generates significant amounts of F&Veg wastes, representing 25–30% of the original mass of the product. The most common wastes include pomace, peel, roots, and seeds. With their high moisture content and organic load, these wastes could cause significant environmental contamination [21,22,23]. In the past, F&Veg wastes have been discarded as food waste due to the lack of appropriate processing technologies that are essential for their effective value addition. However, the fact that F&Veg wastes are a plentiful and affordable source of phenolic compounds has been demonstrated [24]. Among them, phenolic acids (e.g., hydroxybenzoic acid and hydroxycinnamic acid) and flavonoids (e.g., oligomeric tannins) are the two major phenolic components in F&Veg wastes [25]. For example, citrus peel is rich in phenolic acids such as caffeic acid, *p*-coumaric acid, ferulic acid and, sinapic acid, and flavonoids such as naringin and hesperidin [26]. The phenolic compounds in litchi pericarps mainly include catechins, epicatechins, and procyanidins [27]. EPs and NEPs have been subjected to several studies in recent years to reveal their content in F&Veg wastes from materials such as fruit peels and pomace, seeds, and leaves [28,29,30,31]. They extracted and prepared from F&Veg wastes could be reused in the food, cosmetic and pharmaceutical industries, such as the preparation of natural food additives, health food ingredients or active pharmaceutical ingredients, and other applications [13,32,33].

There are many excellent reviews surrounding the antioxidant properties of polyphenols. Gloria Domínguez-Rodríguez [34] summarizes the current knowledge of NEPs and the different extraction methods, providing an update on the purification, isolation, identification, and quantification of plant NEPs; while Jara Pérez-Jiménez et al. [35] provide an overview of the nature of dietary NEPs, their occurrence in the diet, metabolic outcomes and possible effects on human health. Ding et al. [36] summarize the extraction procedures and techniques used to recover NEPs from plant-based by-products, describe the main techniques used to characterize NEPs, and outline their potential food, pharmaceutical, nutraceutical, and cosmetic applications. However, no work has been yet able to systematically summarize information on EPs and NEPs from F&Veg wastes. Our innovation is to divide the complex family of polyphenols into EPs and NEPs according to their extraction properties and to focus on the less commonly studied NEPs. This review will be focused on (1) systematically describing the polyphenols in F&Veg wastes and subdividing them into EPs and NEPs and summarizing them separately; (2) reviewing the antioxidant activity of EPs and NEPs; and (3) elucidating the "structure–activity" relationship between polyphenols and antioxidant activity, using different molecules as entry points. Addressing the above issues not only helps to reduce waste and environmental pollution but also provides ideas and directions for exploring other aspects of F&Veg waste reuse from a commercial perspective. 

## 2. Phenolic Compounds from F&Veg Wastes

### 2.1. Major Phenolic Compounds in F&Veg Wastes

Phenolic compounds can be divided into flavonoids and non-flavonoids, the former including flavonoids, flavonols, flavanones, anthocyanins, flavan-3-ols, etc., and the latter mainly phenolic acids, streptavidin, and diarylheptanoids [37]. According to the phenolic compounds of different F&Veg wastes, Table 1 lists the content and loss of some major phenolic compounds of F&Veg wastes.

Characteristic phenolic compounds are found in different wastes; the green hull of pecan is mainly hickory quinone and coumaric acid [38]. The main flavonoids found in citrus wastes are hesperidin and neritaloside [39]. Sixteen compounds are found in tomato by-products, the concentration of flavonoids was higher than phenolic acids, and the most abundant phenolic acid was caffeic acid [40]. Chlorogenic acid was abundant in vegetable wastes, the main phenolic compounds in broccoli waste were neochlorogenic acid and chlorogenic acid, and chlorogenic acid and quercetin-3-O-rutinoside were predominant in mung bean wastes [41]. Potato peels contain chlorogenic acid, caffeic acid, and ferulic acid [42]. The major phenolics may differ in the by-products, depending on their color. The main ones in the green skin of figs (*Ficus carica* Linn.) were rutin and 5-caffeoylquinic acid, and in the dark purple fig skins were cyanidin-3-O-rutinoside [43]. In eggplant peel, 5-O-caffeoylquinic acid was proven to be the most abundant polyphenol [44]. The color-rich epidermis of F&Veg wastes also tended to contain more anthocyanins [34]. In addition, the differences in the phenolic compounds contained in the different parts of the waste are noteworthy. The residue of the apples presents procyanidins, which are the main NEPs [45]. The seeds are not seen but contain more phenolic acids [46]. If the target extraction is procyanidins, fruit pomace, and peel would be a better source. Unfortunately, in most studies, the mixed phenolic compounds are analyzed in many wastes. If these components could be studied separately, they might be able to be used in a more targeted way.

The phenolic compounds of extractable and non-extractable fractions in F&Veg wastes differ, with some compounds found only in one fraction. To fully characterize the total phenolic compounds in F&Veg wastes, the extractable and non-extractable fractions need to be analyzed separately [34]. Proanthocyanidins (PAs) are the collective name for oligomers and polymers composed of flavan-3-ol monomers, also known as condensed tannins, which are the second most abundant natural phenolic compounds after lignans [47]. In plants, NEPs exist as PA and their flavan-3-ols or hydrolyzed tannins (HTs) from gallic and ellagic acids [48]. Briefly, NEPs are mainly high molecular weight phenolic compounds such as non-extractable proanthocyanidins (NEPAs) and hydrolyzable phenolic compounds (HPPs) bound to macromolecules [18]. As seen in Table 1, flavonoid compounds are present in both EPs and NEPs, as are phenolic acids, which are not all EPs. For example, citrus peels contain high concentrations of bound phenolic acids, which bind to the cell wall of the fruit through ester and glycosidic linkages. Among these, ferulic acid is the main bound phenolic acid, and p-coumaric acid is the main free phenolic acid [26]. Citrus wastes had significantly higher levels of non-extractable gallic acid and catechins compared to those detected in EPs [33]. The situation is also the same with hugger and red kale wastes, where the NEPs fraction is dominated by ferulic and erucic acids, which may be linked to the plant matrix through ester bonds that are cleaved during alkaline hydrolysis [34]. As a high molecular weight phenolic compound, PAs are not only present only in NEPs but also some oligomeric PAs are extracted by solvent to be present in EPs. The mean average degree of polymerization (mDP) of PAs in the EPs fraction of apple pomace is 4.7, and the average DP of PAs in NEPs is eight [45]. Overall, due to the wide structural diversity of the phenolic compound family, the identity of most of the phenolic compounds detected in F&Veg wastes is still uncertain. New structures are still being identified, and more research is needed for this unidentified fraction of phenolic compounds [49].

**Table 1:** Total, extractable, and non-extractable phenolic compounds contents in the waste of different fruit and vegetables.

Commodity	Waste Part	Phenolic Compounds	Extractable Phenolic Compounds Content	Non-Extractable Phenolic Compounds Content	Total Phenolic Compounds Content	Loss Amount/Year	References
**Fruit by-products**							
Apple	Dreg	EPs: (+)-catechin, (–)-epicatechin, quercetin and chlorogenic acid; NEPs: anthocyanidins	25,420 ± 2240, 53,670 ± 2390, 46,470 ± 530, 336,420 ± 14,590 mg/kg DW	184 ± 2.4–234.8 ± 1.5 mg/kg DW	539,840 ± 8900 mg GAE/kg DW	Nearly 30%	[50]
Apple	Pomace	Flavan-3-ols, flavonols, dihydrochalcones, hydroxycinnamic acids, procyanidins	Flavan-3-ols: 2880 mg/kg DW, DP 4.7; Flavonols: 1920 mg/kg DW; Dihydrochalcones:140 mg/kg DW; Hydroxycinnamic acids: 30 mg/kg DW	Procyanidins: 912,000 mg/kg DW, DP 8, and residual amounts of hydroxycinnamic acids (3%), flavonols (2%) and dihydrochalcones (1%)	Hot water: 28,000 60%AC: 78,000 Mix 42,000 Unit: mg/kg DW	Ibid.	[45]
Apple	Seed	Phloridzin, ellagic acid, epicatechin, caffeic acid, catechin, ferulic acid, gallic acid	2861–5141 mg GAE/kg DW	Nd	Nd	Ibid.	[46]
Avocado	Waste water, seeds, peels and pomace	Epicatechin and trans-5-O-caffeoyl-D-quinic acid, procyanidin B1, catechin	Nd	Nd	137, 81, 36, 16 g GAE/kg DW respectively.	18–23%	[51]
Avocado	Peel	Hydroxybenzoic, hydroxycinnamic acids derivatives, flavonoids, catechin, epicatechin, procyanidin, trans-5-O-caffeoyl-D-quinic acid	PP: 63,510 ± 300 mg GAE/kg; Procyanidin B2: 283,000 ± 48,400 mg/kg DW; Epicatechin: 30,400–40,200 mg/kg DW	Nd	Nd	Ibid.	[51]
Banana	Flower, pseudo-stem	Hydroxybenzoic acids, hydroxybenzaldehydes, hydroxycinnamic acids, flavonols	Flower: 10,750.2 ± 206 mg/kg DW	HPP flower: 7352.3 ± 1039.1 mg/kg DW; Pseudo-stem: 1507.5 ± 535.4 mg/kg DW; NEPA flower: 112,000 ± 33,576.1 mg/kg DW	Nd	Nd	[49]
Banana	Inner and outer bracts	Catechin hydrate, chlorogenic, clove, p-coumaric, ferulic, salicylic, quercetin dihydrate and quinic acid Bound phenolics: ferulic acid and salicylic acid	Catechin hydrate (out): 2510 ± 10; Chlorogenic (out), Vanillic (in), Syringic acid (in): 6550 ±320, 1190 ± 60 and 1400 ± 20, respectively; p-coumaric acid: 34,800 ± 170 (out), 6050 ± 100 (in); Ferulic acid: 1860 ± 50 (out), 2790 ± 90 (in); Salicylic acid: 9860 ± 470 (out), 6230 ± 210 (in); Quercetin dihydrate: 5180 ± 600 (out), 1810 ± 90 (in); Quinic acid: 3630 ± 50 (out), 9170 ± 110 (in); unit: mg GAE/kg DW	Ferulic acid: 265,260 ± 730 (out), 141,410 ± 550 (in); Salicylic acid: 95,750 ± 860 (out); 32,920 ± 140 (in); unit: mg GAE/kg DW	7560 ± 110 (out); 9440 ± 50 (in); unit: mg GAE/kg DW	About 300 kg per hectare	[52]
Berry strawberry, raspberry, blueberry, blackberry	Dreg	EPs, EFs, EAs, EPAs, NEPAs, acid and alkaline hydrolyzable phenolic compounds	EPs: 22,310 ± 1140(str), 30,750 ± 1100(ras), 29,430 ± 2130(blu), 28,920 ± 2560(bla) mg GAE/kg; EFs: 3260 ± 200(str), 2910 ± 300(ras), 17,100 ± 1140(blu), 2910 ± 320 (bla) mg RE/kg; EAs: 159.8 ± 1.8(str), 101.4 ± 8.9(ras), 5091.3 ± 140.0(blu), 251.7 ± 9.2(bla) mg C3GE/kg; EPAs: 19,700 ± 780(str), 28,210 ± 1520(ras), 34,330 ± 2260 (blu), 20,660 ± 230 (bla) mg PA/kg	NEPAs: 43,440 ± 2180 (str), 2810 ± 230 (ras), 56,370 ± 2020 (blu), 3730 ± 40 (bla) mg PA/kg; Acid hydroly: 1730 ± 100 (str), 1420 ± 110 (ras), 2030 ± 90 (blu), 3280 ± 320 (bla) mg GAE/kg; Alkaline-hydroly: 2010 ± 110 (str),1420 ±140 (ras), 1680 ± 120 (blu), 1730 ± 150 (bla) (mg GAE/kg)	Nd	20–30%	[53,54]
Buriti	Peels, pulp, endocarp	Free phenols, procyanidins	9346 ± 340 mg GAE/kg	NEPA: 40,853 ± 4740 mg/kg	Nd	8500 tons	[55]
Citrus	Pomace	10 phenolic acids (benzoic), 3 flavonoids (epicatechin), 4 flavanones (hesperidin)	TPC: 21,690 ± 910 mg GAE/kg DW; TFC: 12,870 ± 1070 mg RE/kg DW; CT: 1230 ± 40 mg CE/kg DW	TPC: 74,560 ± 730 mg GAE/kg DW; TFC: 21,560 ± 240 mg RE/kg DW; CT: 476 ± 120 mg CE/kg DW	Nd	15×10^6^ tons	[39]
Cantaloupe Melon	Peels, seeds	Polyphenol, ortho-diphenol, flavonoid, tannin	For peel and seeds: 25,480 ± 1440 and 1500 ± 20 mg GAE/kg; 17,860 ± 1430 and 920 ± 40 mg CAE/ kg; 15,190 ± 1880 and 740± 30 mg CE/kg; 11,830 ± 1440 and 920 ± 30 mg GAE/kg, respectively	Nd	Nd	Peel 25% Seed 7%	[56,57]
Fig	Peel	28 major phenolic compounds: 3 phenolic acids, 3 flavan-3-ols, 8 flavonoids, 6 flavanols, 8 anthocyanins	Rutin: 75,000–200,000 mg/kg DW; Centaureidin-3-O-rutinoside: 2610 mg/kg DW; 5-Caffeoylquinic acid: 131 mg/ kg DW; Total flavan-3-ol: 41 mg/kg DW; Total anthocyanins: nearly 2900 mg/kg DW	Nd	Nd	Nd	[43]
Grape	Pomace	Phenolic acids, Flavonoids, anthocyanins, procyanidins, Flavanols	Procyanidins: 5782 ± 184 EPA mg/kg DW; Anthocyanins: 3600 ± 42 GE mg/kg DW	HPP: 2229 ± 4 GAE mg/kg DW; NEPA: 54,700 ± 2700 mg/kg DW	726 ± 24 mg GAE /kg DW	13–20%	[58,59]
Grape	Dreg	23 flavonoids, 4 hydroxybenzoic acids, 5 hydroxycinnamic acids, phenolic acid hexoxide dihydroxybenzoate is the main	EP: 19,870 ± 1710 mg GAE/kg; EFs: 6100 ± 330 mg RE/kg; EPAs: 9370 ± 620 mg RE/kg	Acid HPPs: 740 ± 60 mg GAE/kg; Alkaline HPPs: 510 ± 40 mg GAE/kg; NEPAs: 1570 ± 70 mg PE/kg	Nd	Ibid.	[60,61]
Hawthorn	Pomace	7 flavonoids (2 flavan-3-ols, 1 flavanone, and 4 flavanols), and 5 phenolic acids (2 hydroxybenzoic acids, and 3 hydroxycinnamic acids)	Nd	Acid hydrolysis TPC: 786.64 ± 6.12 mg GAE /kg; TFC: 556.46 ± 5.73 mg CE/kg; Alkaline hydrolysis TPC: 698.13 ± 9.25 mg GAE /kg; TFC: 486.87 ± 2.76 mg CE /kg; Enzyme-assisted extractionTPC: 729.68 ± 5.53 mg GAE /kg; TFC: 524.09 ± 3.85 mg CE/kg	Nd	30–40%	[62]
Litchi	Pericarp	Flavonoids such as tea phenolic compounds, epicatechin, type A procyanidins and B, rutin, quercetin-3-glucoside and quercetin	TPC: Hydrophilic and Hydrophobic part are 59,000 and 7000 mg GAE/kg DW, respectively; TFC: Hydrophilic and Hydrophobic part: 17,000 and 4000 mg CE/kg DW, respectively	TPC: Hydrophilic and Hydrophobic part: 75,000–99,000 and 10,000–27,000, respectively; TFC: Hydrophilic and Hydrophobic part: 23,000–30,000 and 7000–15,000 mg CE/kg DW, respectively	Total tannin: 1286.58 ± 13.87–1456.12 ± 15.73 mg/kg DW	15%	[63,64]
Lemon	Pomace	Benzoic acid, gallic acid, quercetin rutinoside, naringin, naringenin, naringenin, naringenin,	13,100 ± 1700 GAE mg/kg DW	Nd	Nd	Maximum 60%	[65,66]
Mandarins	Dreg	23 flavonoids, 4 hydroxybenzoic acids, 5 hydroxycinnamic acids, phenolic acid hexoxide dihydroxybenzoate is the main	EPs: 8870 ± 740 mg GAE/kg; EFs: 4120 ± 190 mg RE/kg; EPAs: 5440 ± 520 mg RE/kg	Acid HPPs: 1340 ±50 mg GAE/kg; Alkaline HPPs: 2740 ± 120 mg GAE/kg; NEPAs: 1570 ± 100 mg PE/kg	Nd	50–70%	[60,67]
Mango	Peel	Phenolic compounds	102,800 mg EAG/kg	HPPs: 11,650 ± 310 mg EAG/kg	Nd	25–60%	[68]
Mangosteen	Peel	NEPs: 6 anthocyanins, 4 flavanols, 7 xanthones, and 8 phenolic compounds EPs: 4 xanthones	DMAC: 24 ± 2; Butanol/HCl: 10,170 ± 570 Unit: mg ECE/kg Sample	DMAC: 2380 ± 210; Butanol/HCl: 376,380 ± 63,890 Unit: mg ECE/kg Sample	Nd	Nearly 60%	[29,69]
Oranges	Dreg	23 flavonoids, 4 hydroxybenzoic acids, 5 hydroxycinnamic acids, phenolic acid hexoxide dihydroxybenzoate is the main	EPs: 7860 ± 360 mg GAE/kg; EFs: 3660 ± 150 mg RE/kg; EPAs: 4210 ± 410 mg RE/kg	Acid HPPs: 730 ± 40 mg GAE/kg; Alkaline HPPs: 1810 ± 60 mg GAE/kg; NEPAs: 1430 ± 0.00 mg PE/kg	Nd	35%	[60]
Passiflora	Peel	36 phenolic compounds	TPC:203.6 ± 0.9 mg GAE/g DW; TFC: 753 ± 7 mg CE/g DW	TPC: Acid: 833,000 ± 5000 mg GAE/kg DW; Alkaline: 622,000 ± 2000 mg GAE/kg DW; TFC: Acid 513,000± 61,000 mg CE/kg DW; Alkaline:84,000 ± 15,000 mg CE/kg DW	30,086 mg/kg DW	53%	[70,71]
Passion Fruit	Seed	Stilbene, phenolic acid, flavonoid	Piceatannol and kaempferol: 4800–36,800, 3750 mg/kg DW; Coumarin and p-coumaric acid: 600 mg/kg DW and 96 mg/kg DW	Nd	Nd	Nd	[72]
Peach “Hujingmilu”(Hj), “Dahonghua”(Dh), “Fenghuayulu”(Fh), “Wulingyulu”(Wl)	Peel	Chlorogenic acid, (+)-catechins, neochlorogenic acid, epicatechin, derivatives of centaureidin and quercetin	Cultivation (cv.) Hj, Dh, Fh and Wl): 791.4 ± 48.1, 1014.5 ± 9.5, 1248.8 ± 31.2 and 1671.0 ± 74.2 mg GAE / kg DW, respectively	cv. Hj, Dh, Fh and Wl: 572.2 ± 13.2, 563.4 ± 9.3, 529.3 ± 62.8 and 840.2 ± 84.6 GAE /kg DW, respectively	cv. Hj, Dh, Fh and Wl: 1363.7 ± 58.1, 1577.9 ± 7.1, 1778.2 ± 50.3 and 2511.3 ± 110.5 GAE/kg DW, respectively	Nd	[73]
Peanut	Seed coat	Phenolic acids, ellagic acid, (+) catechin, epicatechin, procyanidin oligomers	Nd	Nd	150 mg CE/g DW	Nd	[74]
Pequi	Peel	Flavonoids, ellagic acid, ethyl gallate and gallic acid	62,520 mg/kg DW	51,090 mg/kg DW	Nd	80%	[75,76]
Plum	Seed	Rutin, gallic acid, syringic acid, epicatechin, caffeic acid, coumaric acid	Coumaric acid: 113.1–129.8 mg/kg DW; Gallic acid: 3.6–5.3 mg/kg DW; syringic acid: 4.5–13.1 mg/kg DW; caffeic acid: 2.9–6.3 mg/kg DW; rutin: 33.4–48.7 mg/kg DW; Epicatechin: 8.9–11.2 mg/kg DW	Nd	1836 ± 46- 2689 ± 78 mg GAE/kg DW	10%	[77]
Pomegranate	Peel	Tannins, phenolic acids, flavonoids NEPs: β-Sitosterol-3-O-glycoside, β-sitosterol, ursolic acid, corosolic acid, asatic acid, arjunolic acid	549,100 mg/kg DW	30.01 ± 0.055 μmol GAE / g DW	Nd	50%	[30]
Prickly pear	Peel Red(r), yellow-orange(y-o), green(g)	68 EPs, (ferulic and benzoic acid, kaempferol 3-O-glucoside); 15 HPPs (gallic acid 3-O-gallate, cinnamic acid, hesperidin)	TPC: 9640 ± 500 (r), 8620 ± 250 (y-o), 12,280 ± 250 (g) GAE mg/kg TFC: 2450 ± 290 (r), 3080 ± 140 (y-o), 3070 ± 160 (g) CE mg/kg	CTs: 710 ± 70(r), 1170 ± 80(y-o), 1080 ± 50(g) PAE mg/kg HPPs: 1180 ± 10(r), 1320 ± 10(y-o), 2010 ± 70(g) GAE mg/kg	9640 ± 500(r); 8620 ± 250(y-o); 12,280 ± 250(g) GAE mg/kg	30–50%	[78,79]
Roselle	Calyces	Hydroxycinnamic acids, procyanidins, hydroxybenzoic acids, flavonols, anthocyanins, organic acids, ellagic acid, quercetin hexoside, ferulic acid	TPC (GAE mg/kg) 6830 ± 180; Flavonoids (CE mg/kg) 5630 ± 460; Anthocyanins: 2470 ± 170 mg C3G/kg	HPs: 6180 ± 80 GAE mg/kg; NEPAs: 6670 ± 30 PAE mg/ kg	Nd	Nearly 50%	[80,81]
Strawberry	Leaf	Ellagic acid, epicatechin, isorhamnetin, tyrosol, quercetin derivate	TPC: 0.47 ± 0.04 mmol GAE/g DW TFC: 18,070 ± 1810 mg CE/kg DW	Nd	Nd	Nd	[82]
Tomato	Seeds, pulp and skins	Simple phenolic compounds, phenolic acids, hydroxycinnamic acids, flavonoids, naringenin	SP: 517.5 ± 70.5, P: 128.1 ± 7.6 H: 120.8 ± 8.3; F: 378.7 ± 62.3 N: 63.5 ± 4.6 unit: mg/kg DW	Nd	9452.8 ± 476.6 mg GAE /kg DW	1–4%	[83]
Sweet cherry	Pomace	Flavonols, flavan-3-ols, anthocyanins, hydroxycinnamic acids, hydroxybenzoic acids	TPC: 380 ± 10 mg GAE/kg DW EPAs: DMAC: 0.2 ± 0.1 mg ECE/kg DW Vanillin: 26 ± 8 mg ECE /kg DW Butanol/HCl: 39 ± 2 mg ECE /kg DW	NEPAs: DMAC: 1.5 ± 0.3 mg ECE/ kg DW Vanillin: 820± 20 mg ECE/kg DW Butanol/HCl: 430 ± 30 mg ECE /kg DW	1870 ± 50 mg GAE/kg DW	3500–7000 tons	[84,85]
Umbu	Peel	p-coumaric, quercetin, ellagic acid, procyanidin B2, syringic acid and protocatechuic acid	Ripe peel: 12,294.3 ± 1253.4 Semi-ripe peel: 15,827 ± 2780.9 unit: mg GAE/kg DW	NEPA (mg/kg DW): R: 9018.9 ± 1409.1, S: 15,446 ± 2965.8 Hydrolyzable tannins (mg TAE/kg DW) R: 13,484 ± 198.6, S: 10,797 ± 433.1	Nd	Nd	[86]
**Vegetable by-products**							
Black radish	Peel	Epicatechin, hydroxybenzoic acid gallic acid, ferulic acid, chlorogenic acid, sinapic acid, syringic acid	E: 19,820 ± 3600; H: 2160 ± 140 G: 7320 ± 1440; F: 28,020 ± 4500 C: 3300 ± 140; Si: 11,150 ± 650 Sy: 2020 ± 70, unit: mg /kg DW	Nd	305,510 ± 515 mg GAE/kg DW	Nd	[87]
Bottle gourd Ridge gourd	Peel	phenolic acids, phenolic acid glycosides, terpenoids, flavonoids, etc.	91,000 mg GAE/kg DW 23,000 mg GAE/kg DW	Nd	Nd	Nd	[88]
Carrots	Peels	Hydroxybenzoic acid, gallic acid, chlorogenic acid, caffeic acid, ferulic acid, p-coumaric acid	8300 ± 600 GAE mg/kg DW	Nd	Nd	11%	[65,89]
Cauliflower	Peels, leaves, and stems	Phenolic compounds	TPC: 2230–3450 mg GAE/kg DW TFC: 240–350 mg CE/kg DW	Nd	Nd	Nd	[90]
Fennel	Bulbs, stems, flowers external leaf sheaths.	Quercetin, chlorogenic acid, caffeic acid, ferulic acid, p-coumaric acid	5570 ± 920 GAE mg/kg DW	Nd	Nd	Nd	[65]
Garlic	Peels, leaves, and stems	Phenolic compounds	TPC: 4800–6470 mg GAE/kg DW TFC: 620–830 mg CE/kg DW	Nd	Nd	Nd	[90]
Onion	Peels, leaves, and stems	Phenolic compounds	TPC: 12,870–16,120 mgGAE/kg DW TFC: 1720–2130 mg CE/kg DW	Nd	Nd	Nd	[90]
Pea	Pod	5-caffeoylquinic acid, hesperidin, (–)-epicatechin	598.7, 199.4, 294.6 mg/kg, respectively	Nd	TPC: 625.2 ± 6.8 mg/kg DW TFC 214.2 ± 11.3 mg/ kg DW	35–40%	[91]
Red cabbage Brussels sprout	Waste	Phenolic acid derivatives, flavonoid glycosides, acylated flavonoid glycosides, glucosinolates and anthocyanins	3700 ± 0.0 mg GAE/kg DW Top: 1500 ± 0.0 mg GAE/kg DW Stalks: 2000 ± 0.0 mg GAE/kg DW	11,500 mg GAE/kg DW Top: 4800 ± 1200 mg GAE/kg DW Stalks: 3300 ± 200 mg GAE/kg DW	Nd	30%	[34]
Tomato	Peels, seeds, rotten, unripe pieces	Quercetin rutinoside, Kaempferol rutinoside	924 ± 7 GAE mg/kg DW	Nd	Nd	6000 tons	[40,65]

NEPs, non-extractable phenolic compounds; EPs, extractable phenolic compounds; EFs, extractable flavonoids; EAs, extractable anthocyanins; EPAs, extractable proanthocyanidins; NPAs, non-extractable proanthocyanidins; acid and alkaline HPs, acid and alkaline hydrolyzable phenolic compounds; GAE, gallic acid equivalent; CE, catechin equivalents; ECE, epicatechin; DW, dry weight of defatted sample; CT, concentrated tannins; DP, degree of polymerization; TPC, total polyphenol content; TFC, total flavonoid content; HPP, hydrolyzed phenolic compounds; Nd, not detected; butanol/HCl, vanillin and DMAC are different methods to test the total proanthocyanidin content; Ibid., in the same place.

### 2.2. Content of Major Phenolic Compounds in Wastes

The inedible parts of F&Veg generally account for a large portion of the overall part. Table 1 provides a rough summary of the annual generation of various F&Veg wastes. However, vegetable wastes are less reported, while the limited edible parts of most fruits are more reported. For example, the peel and seeds of rambutan account for almost 50% of the whole fruit weight [92]. It has also been demonstrated that inedible parts, such as the peel, or rind portion of the fruit typically contain more bioactive components (e.g., phenolic compounds) com-pared to the edible part of the fruits [93]. Many fruits, e.g., pears, blueberries, apples, citrus, and mangoes, contain more phenolic compounds in their peel tissues compared to their flesh [26,73]. For example, the phenolic compound contents are varied among different wastes; the peel extracts of two selected avocados exhibited higher phenolic content (63.5 and 120.3 mg/g, respectively) than the seed extracts (57.3 and 59.2 mg/g, respectively) [51].

Combined with the summary of the EPs and NEPs contents of different F&Veg wastes in Table 1, the phenolic contents of F&Veg wastes were considerable. In apple pomace [50], the content of chlorogenic acid was very high, with 336.42 ± 14.59 mg/g DW. Onion is the second most important vegetable crop after tomato. Red onion peel was rich in phenolic compounds, e.g., flavonoids, ranging from 1.276 mg/g to 169 mg/g [94]. Red/green currant and sea buckthorn berry press also contained high amounts of phenolic compounds. Unfortunately, only the total phenolic and non-phenolic contents are determined in the study without specifying the EPs and NEPs contents [95]. In some F&Veg wastes, the content of NEPs was higher than that of EPs. For example, mangosteen peel had a much higher content of NEPs than EPs [29], and the content of extractable proanthocyanidins (EPAs) in carrion pomace was lower than that of NEPAs [84]. Most of the current studies on F&Veg wastes have investigated EPs, with NEPs accounting for only a very small fraction of them [17]. Most of the studies characterizing fruits and their by-products did not determine the content of NEPs, ignoring their contribution to the total polyphenol content [78]. In addition, extractable phenols decreased with the advancement of the maturation process [86]. This is probably because EPs play a greater role in the function of protecting plants from external aggressions such as bacteria and insects due to their free state. Therefore, the content of NEPs in F&Veg wastes may account for a larger fraction of the total phenolic content.

## 3. The Antioxidant Properties of Extractable and Non-Extractable Phenolic Compounds

Phenolic compounds, due to the high reactivity of hydroxyl substitution in their structure and their ability to engulf free radicals, give them the potential for antioxidant activity [26]. Similarly, phenolic compounds found in F&Veg wastes have antioxidant properties and could act as reactive oxygen scavengers and free radical inhibitors [90]. The peel, one of the F&Veg wastes, is rich in phenolic compounds and antioxidants, so they could protect fruits from oxidative stress caused by sunlight and high temperatures [96]. The phenolics present in the waste are generally in both free and bound forms, and the antioxidant properties of the two types of phenolic compounds differ (Table 2).

### 3.1. Antioxidant Properties of Extractable Phenolic Compounds

The antioxidant properties of extractable phenols, which can be easily recovered with water or aqueous organic solvents, have been extensively studied. For example, 80% ethanol extracted a large number of EPs. In addition, the combination of different polar solvents (e.g., methanol, acetone, and water) facilitated the extraction of flavonoids and increased their antioxidant capacity [97,98]. Most of the studies on the antioxidant properties of phenolic compounds in F&Veg wastes refer to EPs [62]. As shown in Table 2, many EPs in F&Veg wastes have better antioxidant properties. For example, the EC50 of fruit Cantaloupe Melon peel against DPPH radicals was 6.65 mg/mL [56]. The antioxidant capacity of vegetable red pepper and cucumber wastes was also high, with 2.34 ± 0.14 mmol Trolox/100 g DW in the ABTS test and 7.00 ± 0.51 mmol Fe^2+^/100 g DW in the FRAP test, respectively [99]. Moreover, the antioxidant properties of EPs are affected by several factors. Differences in antioxidant capacity of phenolics in different wastes of the same F&Vegs. As an example, the main wastes of longan were peels and seeds, and the scavenging of DPPH radicals was better for the former in terms of ORAC values seed phenolics (7750.8 ± 1135.6 μmol TE/g) and peel phenolics (6868.2 ± 386.2 μmol TE/g) [100]. Avocado had the best antioxidant capacity of extractable phenolic compounds in the peel, followed by pomace and seeds [51]. The antioxidant capacity of EPs in different species of Passion Fruit was also highly variable, with P. ligularis having better antioxidant capacity in both DPPH and ABTS tests with IC_50_ values. Moreover, the EPs extracted from different organic solvents also affect its antioxidant properties, e.g., apple pomace in 60% Acetic acid had the best EPs antioxidant properties [45]. Blanching is an important step in the extraction of EPs from fruits and vegetables during processing and is a treatment method that facilitates the antioxidant properties of EP. This is because the presence of certain enzymes (including polyphenol oxidase) in fruits and vegetables can inhibit antioxidant effects [55]. It can be affirmed that F&Veg wastes are relatively rich in free phenolics, which makes the simple extraction also makes it exhibit good antioxidant capacity.

### 3.2. Antioxidant Properties of Non-Extractable Phenolic Compounds

Non-extractable phenolic compounds are derived from food residues that have already undergone simple extraction with solvents due to binding interactions with other biological substances [48]. If secondary extraction of NEPs from this fraction is desired, the most commonly used methods are alkaline hydrolysis, acid hydrolysis, and enzymatic hydrolysis. Alkaline hydrolysis breaks ether and ester bonds; acid hydrolysis breaks glycosidic bonds; enzymes break hydrophobic or hydrogen bonds to extract [62]. Acid-base hydrolysis is simple and rapid, but some phenolic compounds are unstable to extreme pH values [101]. Enzyme-assisted hydrolysis enhances NEPs release through specific hydrolysis and requires complex enzyme manipulation for adequate hydrolysis [102]. The extraction method during this process affects the phenolic content and composition of NEPs [62]. Due to the complexity of NEPs, it is also necessary to separate NEPs in fruit samples by HPLC, high-speed counter-current chromatography (HSCCC), or high-performance thin-layer chromatography (HPTLC) [103].

NEPs are mainly high molecular weight polymeric phenolic compounds or individual low molecular weight phenols chemically linked to macromolecules, e.g., cell walls (e.g., cellulose and hemicellulose complexes) and peptide networks [48]. Among these low molecular weight phenolic compounds are hydrolyzable phenolic compounds, most commonly phenolic acids (e.g., ferulic acid, caffeic acid, erucic acid). In addition, most NEPs are proanthocyanidins, which are associated with high molecular weight phenolic compounds or macromolecular, e.g., proteins or dietary fibers [35]. It is worth noting that NEPs mainly include phenolic compounds, also found as EPs compounds, e.g., proanthocyanidins, other flavonoids, phenolic acids, and HTs [35]. 

The phenolic content and bioactivity of NEPs fruit residues may be underestimated if neglected. As shown in Table 2 for the antioxidant capacity of phenolic compounds in F&Veg wastes, it is clear that the antioxidant properties of extractable phenolic compounds have been studied more than those of non-extractable ones in F&Veg wastes. Some of the studies concluded that extractable phenolics contribute more to antioxidant activity compared to non-extractable ones [104]. However, with the increased understanding of NEPs, more and more studies have demonstrated that the phenolic content and antioxidant capacity of NEPs fractions are higher than that of EPs fractions [28]. The antioxidant capacity of NEPs in apple pomace is 3–4 times higher than that of EPs [50]. In litchi pericarps [63], although the contribution of extractable antioxidants to the total antioxidant activity was 38–65%, the contribution of non-extractable compounds was greater. The same was observed in citrus fruit pomace [39], where the combined data of four antioxidant tests showed that the non-extractable’s had more prominent antioxidant properties. There may be other components with reducing activity, metal catalysts, or synergistic effects of phenolics in the waste that affect the antioxidant activity of soluble and bound phenolics [105], and the presence of catechins in bound phenols of apple peels may positively affect their antioxidant activity compared to apple pomace. However, peach peel showed the opposite conclusion [73], as the phenolic content could be extracted more, and a direct relationship between total phenolic content and antioxidant or free radical scavenging activity was not necessarily found [105], but in general, samples with higher phenolic content were effective free radical scavengers [65].

In general, F&Veg wastes can be underestimated to some extent by neglecting the study of NEPs, which should be added when measuring the antioxidant capacity of the wastes. While isolating the two phenolic compounds in the wastes in the study, assessing their contribution to the total antioxidant capacity can identify the secondary or primary contributors to the antioxidant properties, which can provide a fuller understanding of the antioxidant properties of F&Veg waste to facilitate better utilization of resources [106]. Therefore, it is important to focus on the study of NEPs in F&Veg wastes. In addition, current studies on the antioxidant properties of EPs compounds have found that solvents, species, waste types, and enzymes in the matrix affect the antioxidant properties, which could also be explored in the study of NEPs.

**Table 2:** The antioxidant properties of extractable and non-extractable phenolic compounds present in F&Veg wastes.

Commodity	Waste Part	Measurement Items	Antioxidant Properties of Extractable Phenols	Antioxidant Properties of Non-Extractable Phenols	References
**Fruit by-products**					
Apple	Dreg	DPPH ABTS	(40 μg sample) 16.20 ± 2.29% Over 30%	(40 μg sample) 80.54 ± 3.23% Nearly 100%	[50]
Apple	Seed	ABTS DPPH	292–392 μmols TE/g DW(ABTS) 21.5–43.6 μmols TE/g DW (DPPH)	Nd	[46]
Apple	Pomace	ABTS	Hot water: b33, 60%AC: 95, Mix: 23, Unit: mmol TE/kg	Nd	[45]
Avocado	Waste water, seeds, peels and pomace	CUPRAC FRAP PMB	Unit: g TE/100 g powder Seed: 2.1 ± 0.09, 7.7 ± 0.35, 8.1 ± 1.00 Pomace: 7.1 ± 0.10, 4.0 ± 0.01, 6.8 ± 1.90 Skin: 7.0 ± 0.12, 13.7 ± 0.76, 12.0 ± 1.19 Wastewater:3.3 ± 0.87, 2.0 ± 0.19, 3.2 ± 0.4	Nd	[51]
Banana	Inner and outer bracts	DPPH ABTS FRAP	Unit: μmol TE/g fresh mass DPPH: outer: 24.53 ± 0.04, inner: 27.96 ± 0.11, ABTS: outer: 29.62 ± 0.10, inner: 30.66 ± 0.15 FRAP: outer 8.85 ± 0.04, inner 20.6 ± 0.45	Nd	[52]
Buriti	Peels, pulp, endocarp	DPPH FRAP	IC50: 413.1 ± 14.9 (mg/g DPPH) 155.5 ± 4.6 (μmol Fe_2_SO_4_/g)	Nd	[55]
Cantaloupe Melon	Peels and seeds	DPPH FRAP	EC50: 6.65 (p), 55.03 (s) mg/mL 12.27 ± 1.22 (p), 0.31 ± 0.02 (s) mg AAE/g	Nd	[56]
Citrus	Pomace	ABTS ORAC DPPH FRAP	574.71 ± 19.25 μM TE/g DW 196.02 ± 7.91 μM TE/g DW 140.82 ± 2.72 μM TE/g DW 50.34 ± 0.63 μM TE/g DW	549.55 ± 19.54 μM TE/g DW 589.63 ± 13.78 μM TE/g DW 351.55 ± 3.73 μM TE/g DW 10.84 ± 0.52 μM TE/g DW	[39]
Custard Apple	Seed, peel	FRAP TEAC ORAC HOCL NO	S:0.292 ± 0.005, P: 0.27 ± 0.01 mmol Fe^2+^/g DE S: 171 ± 2, P: 130.0 ± 0.4 μmol TE/g DE) S: 0.368 ± 0.005, P: 0.324 ± 0.009 (mmol TE/g DE) S:11 ± 2 28 ± 4 O_2_^−^ (mg/L) S: 11.5 ± 0.2, p:11.8 ± 0.3 (mg/L)	Nd	[107]
Date	Seed	DPPH ABTS ORAC	EC50:0.37/0.34 (mg/L) 3.28 ± 0.09 / 6.61 ± 0.15 (mmol TE/g DW) 9.91 ± 0.84/12.82 ± 0.58 (mmol TE/g DW)	Nd	[108]
Grape	Pomace	NO, DPPH ABTS	IC50: 225 μg/ mL, 250μg/ mL, 625 μg/ mL, respectively	Nd	[58,59]
Lemon	Pomace	TEAC DPPH ABTS	16.8 ± 0.2 AAE mg/g DW IC50: 97.3 ± 4.1 μg/mL IC50: 80.2 ± 1.9 μg/mL	Nd	[65]
Litchi	Pericarp	DPPH	Hydrophilic PC 65% Hydrophobic PC 38%	Hydrophilic PC 68–85% HydrophobicPC 70–85%	[63]
Longan	Peels and seeds	DPPH	seed: 7750.8 ± 1135.6 μmol TE/g peel: 6868.2 ± 386.2 μmol TE/g	Nd	[100]
Mango	Peel	ABTS FRAP	ABTS (mmol TE/g) 1293.65 ± 9.05 FRAP (mmol TE/g) 735.68 ± 6.99)	Nd	[51]
Passiflora	Peel	DPPH ABTS	SC50:6.3 ± 0.5 mg TE/mL SC50:3.17 ± 0.09 mg TE/mL	SC50: 5.26 ± 0.03–0.38 ± 0.08 mg TE/mL SC50: 9.8 ± 0.1–2.062 ± 0.007 mg TE/mL	[71]
Passion Fruit	Seed	DPPH ABTS	P. edulis, P. tripartita, P. ligularis, and P. pinnatistipula IC50: 2.7–132.6, 3.2, 73.9, and 372.2, respectively IC50: 9.0, 96.2, 23.9, and >1000, respectively	Nd	[72]
Peach	Peel	TEAC FRAP DPPH	393.1–946.1μmolTE/100 g FW 1.29–2.96 mM FeSO4/100 g EC50 11.7–34.0 mg DW/mL	52.2–83.1 μmolTE/100 g FW 0.42–0.95 mM FeSO_4_/100 g EC50 18.7–25.1 mg DW/mL	[73]
Plum	Seed	TEAC	IC50 (mg/mL) Ethyl-acetate 0.40 ± 0.01 Chloroform: Methanol (2:1 *v*/*v*): 0.65 ± 0.02	Nd	[77]
Strawberry	Leaf	FRAP DPPH ABTS	FRAP: 0.55 ± 0.04 Mmol TE/g DW. DPPH: 0.52 ± 0.03 Mmol TE/g DW. ABTS: 0.78 ± 0.08 Mmol TE/g DW.	Nd	[82]
Sweet cherry	Pomace	·OH DPPH TEAC	5 ± 1% EC 50: 755 ± 36 μg/mL sample 2.6 ± 0.1 μmol Trolox/g sample	94.6 ± 0.2% EC50: 1311 ± 20 μg/mL 50 sample 14.8 ± 1.6 μmol Trolox/g sample	[84]
**Vegetable by-products**					
Asparagus	Around half of the total spear length	DPPH POIC FRAP	EC50: 20.20–31.43 mg of dry extract EC50: 3.92–4.36 mL of solution PR:0.12–0.21 mg of quercetin/mL	Nd	[109]
Black radish	Peel	CUPRAC DPPH FRAP	172.29 ± 11.5 mg TE/g DW 462.72 ± 3.05 mg TE/g DW 796.51 ± 10.04 mg TE/g DW	Nd	[87]
Bottle gourd	Peel	DPPH	Nd	54.23% (50 μL sample) BHT: 77.12%	[88]
Carrots	Peels and discarded material	TEAC DPPH ABTS	5.21 ± 0.8 AAE mg/g DW IC50: 74.4 ± 1.3 μg/mL IC50: 77.1 ± 0.7 μg/mL	Nd	[65]
Fennel	Bulbs, stems, flowers, external leaf sheaths.	TEAC DPPH ABTS	4.97 ± 0.95 AAE mg/g DW IC50: 70.2 ± 0.9 μg/mL IC50: 76.3 ± 0.8 μg/mL	Nd	[65]
Fennel	Waste	DPPH ABTS FRAP	DPPH: 14.2 ± 0.9 mmol/kg ABTS: 17.7 ± 1.4 mmol/kg FRAR: 12.3 ± 1.1 mmol/kg	Nd	[110]
Onion, Garlic Cauliflower	Peels, leaves, and stems	DPPH	methanol extracts: 43.98–57.37% ethanol extracts: 47.28–61.13%	Nd	[90]
Pea	Pod	DPPH ABTS FRAP	IC 50: 1430 ± 20 μg/mL IC50: 1700 ± 10 μg/mL 75 ± 5 μM TE/mg	Nd	[91]
Peanut	Seed coat	DPPH ABTS ·OH	2442 ± 36.5 μmol TE/g DW 1450 ± 15.5 μmol TE/g DW 668.3 ± 5.64 μmol TE/g DW	Nd	[74]
Ridge gourd	Peel	DPPH	Nd	54.23% (50 μlsample) BHT:77.12%	[88]
Tomato	Peels, seeds, rotten, unripe pieces	TEAC DPPH ABTS	1.59 ± 0.11 AAE mg/g DW IC50: 9.23 ± 1.15 μg/mL IC50: 59.0 ± 1.6 μg/mL	Nd	[65]
Tomato	Seeds, pulp and skins	DPPH FRAP	2990.4, 164.4 mMol TE/kg DW 953.7, 77.9 mMol AAE/kg DW	Nd	[83]

CUPRAC, the cupric reducing antioxidant capacity; DPPH, 1,1-diphenyl-2-picrylhydrazyl; FRAP, ferric ion reducing antioxidant power; TE, Trolox equivalents; PMB, phosphomolybdenum blue; SC_50_, concentration of the sample that decreases the initial; POIC, primary oxidation inhibition capacity; AAE, ascorbic acid equivalent.

### 3.3. Non-Extractable Polyphenols Improve Oxidative Stress in the Intestine

In the gastrointestinal digestive system, NEPs are effective in mitigating cellular damage caused by oxidative stress and alleviating damage to the structural morphology of the digestive tract mucosa [111,112]. The intestine is the most important organ in the body, responsible for the digestion and absorption of dietary nutrients on the one hand and playing an important role as a barrier function on the other [113]. The intestine is the front line of immune defense and is vulnerable to damage by exogenous and metabolic oxygen free radicals, inducing oxidative stress in the intestine. Oxidative stress disrupts the tight junctions between intestinal epithelial cells through various pathways, leading to impaired intestinal epithelial barrier function as well as increased intestinal permeability, predisposing to the development of diarrhea, fecal blood, and enteritis, among others [114,115,116]. Nowadays, as people’s living standards continue to improve, high-fat, high-sugar, and high-protein diets are becoming the norm in most people’s lives, and the incidence of intestinal diseases is increasing year by year, the search for healthy, natural alternatives with fewer side effects and better therapeutic effects is becoming a new trend [117]. As a strong natural antioxidant, NEPs are gradually gaining attention. Due to the binding to the plant cell wall, only a small proportion of the active ingredients of NEPs may be released during digestion in the mouth, stomach, and small intestine, where they are absorbed by the body and act as antioxidants in the small intestine. In other words, NEPs can maintain a relatively intact structure in the intestine, remain chemically stable, and have a well-regulated effect on gastrointestinal digestion and colonic fermentation processes. Upon reaching the small intestine, NEPs are fermented and extensively transformed by the colonic microbial community there. On the other hand, EPs are susceptible to structural damage during digestion and absorption, and, are therefore, less biologically active and less available in the human body [15,118,119,120,121]. Therefore, NEPs can either be extracted from food and utilized; or they can be pretreated to make them more readily hydrolyzable in the human body for release and absorption so that NEPs can exert their biological activity in the human body and improve intestinal diseases in humans.

Many studies have been conducted on NEPs to improve intestinal health. Maurer et al. [122] obtained NEPs from grape skins and found that they could reduce inflammation and oxidative responses in experimental colitis in rats by modulating the nuclear factor-κB (NF-κB) pathway and the activity of antioxidant enzymes, reducing nitric oxide (NO) levels and expression of pro-inflammatory cytokines in rats. Huang et al. [123] found that proanthocyanidins extracted from peanut skins could ameliorate dextran sulfate sodium-induced ulcerative colitis (UC) by mediating the intestinal barrier, expression of inflammatory cytokines (TNF-α, IL-β, IL-6, and IL-10) and oxidative stress (MDA, T-SOD, NO, and iNOS) in mice and that due to the intervention of proanthocyanidins, the intestinal microbiota was optimized with increased abundance of *Lachnospiraceae_NK4A136_group*, *Oscillibacter*, and *Roseburia*, and decreased abundance of *Bacteroides*, *Helicobacter*, *Parabacteroides*, *Escherichia-Shigella*, and *Enterobacter* decreased. The metabolome of colonic tissues was significantly altered, as reflected in the modulation of taste transduction, mTOR signaling pathway, PI3K-Akt signaling pathway, and FoxO signaling pathway to improve resistance to UC. NEPs mainly contain flavonols, flavanols, and phenolic acids, which have preventive effects on intestinal inflammation and colon cancer by improving the activity of antioxidant enzymes, balancing inflammatory factor levels, and regulating intestinal flora, among other mechanisms to improve intestinal oxidative stress and impaired intestinal barrier function, but there are complex interactions between the mechanisms that need to be further investigated [47].

## 4. Antioxidant and Structure–Activity Relationships

EPs generally contain free phenolic acids, stilbene, lignans, and some flavonoids, whereas NEPs contain condensed tannins, also called proanthocyanidins, flavonols and, bound phenolic acids, such as chlorogenic acid, ferulic acid, and gallic acid. As phenolic compounds are defined as aromatic compounds with at least one hydroxyl group [124]. The groups attached to the aromatic ring by replacing the hydrogen on the ring are called substituents, and the position and number of different substituents on the ring can modulate the antioxidant activity of phenolic compounds, especially their hydrogen donor capacity. 

### 4.1. Hydroxyl Groups

**Figure 1:**
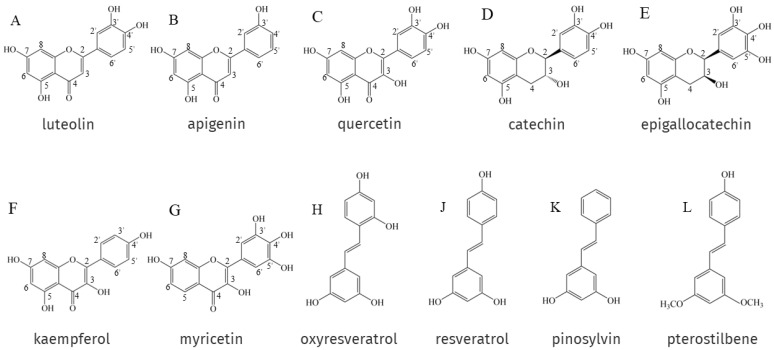
Structural formulae for EPs and NEPs as described in Section 4.1.

The antioxidant activity of EPs and NEPs depends on their chemical structure, especially on the arrangement and number of hydroxyl groups attached to the aromatic ring and the nature of the substituents on the aromatic ring. Phenolic hydroxyl groups can provide oxygen atoms to pair single electrons in the radical structure to reduce the number of single electrons and scavenge DPPH• and ABTS+• [12,125,126]. In general, the higher the number of hydroxyl groups attached to the aromatic ring of phenolic compounds, the better the antioxidant activity. For example, luteolin (Figure 1A) has two ortho-hydroxyl groups on its B-ring, making it a better electron donor than apigenin (Figure 1B), which has only one hydroxyl group. This means that luteolin possesses a more powerful ability to scavenge stress-induced free radicals than apigenin [127]. In addition, Rodríguez-Bonilla et al. [128] showed through in vitro studies that the antioxidant activity of four stilbene compounds, resveratrol (Figure 1J), oxyresveratrol (Figure 1H), pinosylvin (Figure 1K) and pterostilbene (Figure 1L), is closely related to the number of their hydroxyl groups, so oxyresveratrol, which has four hydroxyl groups. It has the most powerful antioxidant and free radical scavenging activity of the four, and the lowest is pterostilbene, which has only one hydroxyl group. However, two polyphenols with the same number of hydroxyl groups do not necessarily have the same antioxidant capacity, and catechin (Figure 1D) possesses the same number of -OH groups as quercetin (Figure 1C); its antioxidant activity is significantly lower. This is due to the fact that catechin has no unsaturated bond at the C2-C3 position to the oxo (-C=O) on the C ring, which is relatively higher in antioxidant activity than quercetin. Epigallocatechin (Figure 1E) has an additional -OH group on the B ring on top of catechin, and the antioxidant activity is improved under this new structure [129]. In addition to the number of hydroxyl groups, the antioxidant activity of EPs and NEPs is also related to the position of the hydroxyl groups on the aromatic ring and the substituents. Flavonols, in NEPs, whose heterocycles promote antioxidant activity mainly through (i) the presence of free 3-OH in the A ring; and (ii) allowing for conjugation between aromatic rings. Firstly, the ability of flavonols to scavenge free radicals is highly dependent on free 3-OH, and luteolin, which lacks 3-OH, is a quite weak scavenger of DPPH (2,2-diphenyl-1-picrylhydrazyl radical) compared to flavonols such as quercetin, kaempferol (Figure 1F) and myricetin (Figure 1G) [130]. Substitution of 3-OH with methyl or glycosyl groups completely eliminates the activity of quercetin and kaempferol against the oxidation of β-carotene in linoleic acid [131]. Free 3-OH is also thought to increase the stability of the flavonol structure. As the torsion angle of the B-ring relative to the rest of the molecule strongly affects the free radical scavenging ability of flavonoids. Flavonols with 3-OH, are, therefore planar, whereas flavones and flavanones lacking this feature are slightly twisted. Planarity allows for conjugation, electronic misalignment, and a corresponding increase in the stability of flavonoid phenoxy radicals [132]. Removal of 3-OH results in the loss of coplanarity and conjugation, which affects free radical scavenging capacity [132,133].

### 4.2. Methyl Groups

**Figure 2:**
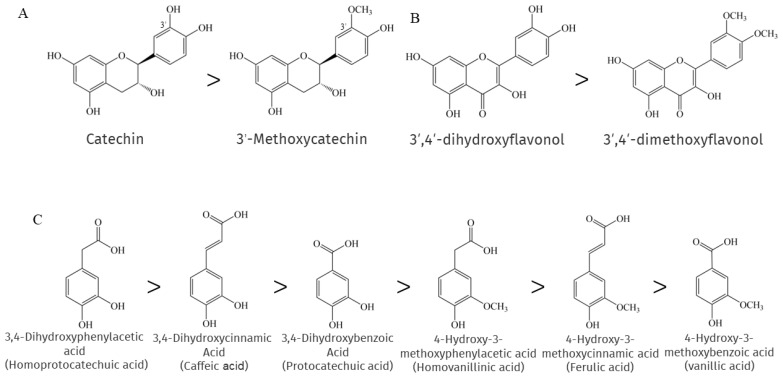
Comparison of the magnitude of the antioxidant capacity of some NEPs and their methylated compounds.

When researchers compared a number of phenolic compounds, including flavonoids and phenolic acids, with their methylated forms in chemical antioxidant capacity assays, they found that methylation largely eliminated the "chemical" antioxidant capacity [134]. This is because methylation leads to a reduction in reactive electron and hydrogen donor groups, resulting in a decrease in the efficiency of phenolic compounds as antioxidants. Su et al. showed that the methylation of 3′-OH on the catechin B ring leads to a significant loss of its antioxidant activity [135], as shown in Figure 2A, and consistent with the findings reported by [136]. A similar situation is found with flavonols belonging to the NEPs, where the 3′- and 4′-OH methylation of flavonols greatly impairs the ability to scavenge Xanthine/xanthine oxidase and pyrogallol-induced superoxide production, as shown in Figure 2B [137]. Interestingly, the methylation of cinnamic acid derivatives, which are also NEPs, had no significant and direct effect on the antioxidant activity, as 4-hydroxy-3-methoxycinnamic acid exhibited slightly lower activity than 3,4-dihydroxycinnamic acid. In contrast, the antioxidant activity of the methylation products of both benzoic acid derivatives and phenylacetic acid derivatives was much lower than that of the parent compound, as shown in Figure 2C, and it seems that the farther the carboxyl group is from the benzene ring, the more effective is the antioxidant capacity of the methylated phenolic acid. This can be explained by the importance of carboxylation-induced effects [138].

### 4.3. Glycosyl Groups

The antioxidant activity of glycosylated phenolic compounds is mainly related to the glycosyl site, glycosyl type, and glycosyl number. Plumb et al. reported that the antioxidant properties of flavonol glycosides in tea decreased with the increase in the number of glycosyl groups [139]. Glycosylation causes a significant decrease in the antioxidant activity of phenolic compounds, especially when the substituent is located at 3-OH, which confers antioxidant activity to the phenolics [140]. The substitution of 3-OH with Glycosyl groups completely eliminated the activity of quercetin and kaempferol on the oxidation of β-carotene in linoleic acid [131]. Differences in glycosylation inevitably alter the antioxidant activity of phenolic compounds, such as free radical scavenging efficiency. Anthocyanidins are found in plants in glycosylated form and are named anthocyanins, the structural composition of several common anthocyanins is summarized in Table 3. The prevalent glycosylated groups are glucose, rhamnose, xylose, galactose, arabinose and, fructose. Both mono- and di-glycosides are common, as well as acylated forms. Glycosyl groups can be located on carbon 3, 5, 7, 3′, and 5′, with the major ones being the 3 and 5 positions. The free radical scavenging efficiency of cyanidin, peonidin and malvidin galactosides was shown to be 15–23% weaker than their corresponding glucosides, and the activity of cyanidin and delphinidin rutinosides was also lower than their glucosides. Again, for the antioxidant activity of monoglucosides, there was no significant difference between glucosides and galactosides, but the free radical scavenging efficiency of arabinosides was significantly lower [140,141,142].

Interestingly, there have been studies on the application of deglycosylation to practical production to improve the antioxidant capacity of fruit products. Silva et al. [143] used enzymatic deglycosylation to convert 60% of hesperidin in orange juice or lemon juice to its aglycone form, hesperetin, thereby improving the antioxidant capacity of orange juice or lemon juice. 

### 4.4. Carbon-Carbon Double Bond

**Figure 3:**
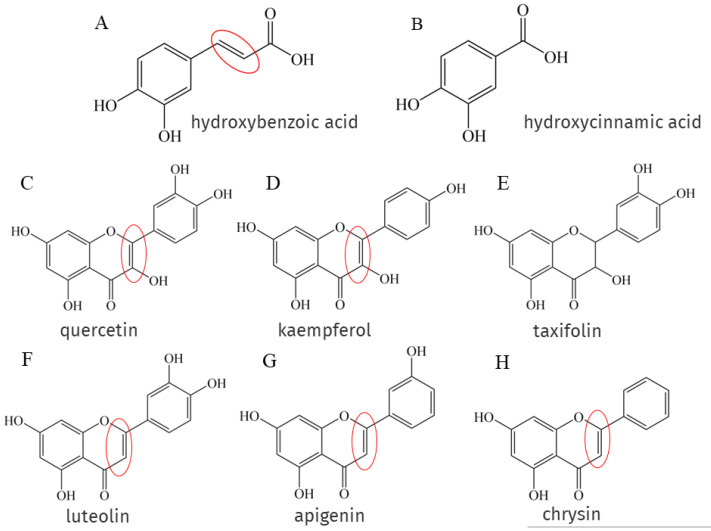
The characteristic double bonds of hydroxycinnamic acid and some flavonoids are specially marked with red circles.

Phenolic acids are the main components of NEPs, and phenolic acids are classified as hydroxybenzoic acid (Figure 3A) and hydroxycinnamic acid (Figure 3B), but the presence of the -CH=CH-COOH group in hydroxycinnamic acid ensures greater antioxidant capacity than the -COOH group in hydroxybenzoic acid because the -CH=CH-COOH structure attached to the benzene ring forms a conjugate with the double bond on the benzene ring, enhancing its ability to stabilize free radicals [20], this structure has been marked with a red circle in Figure 3. There is also a part of flavonoids such as flavonols like quercetin (Figure 3C), kaempferol (Figure 3D), flavones like luteolin (Figure 3F), apigenin (Figure 3G), chrysin (Figure 3H), etc. A remarkable feature of the structures of the above phenolic compounds is the presence of unsaturated 2–3 double bonds when conjugated with the 4-oxo function, which have been marked with red circles in Figure 3. Quercetin and taxifolin (Figure 3E) are structurally similar in that both structures have a 4-oxo group, but taxifolin is saturated between C2 and C3. Experiments have shown that quercetin inhibits ferrous sulfate-induced MDA formation more strongly than taxifolin [132].

## 5. Conclusions and Perspective

In recent years, there has been an increasing demand for the recovery of bioactive substances from F&Veg wastes, with attention focused on improving the entire process from polyphenol extraction to final application, but for the wide variety of F&Veg wastes, it is necessary to analyze and classify them in a detailed and specific way to collate important information such as phenolic content, type and important information on the association between antioxidant properties and their structure. NEPs are also present in high levels in some wastes compared to EPs. Their antioxidant activity was also evaluated by a comprehensive antioxidant assay, and both EPs and NEPs were found to have high antioxidant activity. However, as an essential phenolic component found mainly in plant-based foods, NEPs have not been adequately considered in polyphenol studies of F&Veg wastes. It is, therefore, particularly important to understand all aspects related to non-extractable polyphenols in F&Veg wastes. In addition, the number and position of phenolic hydroxyl groups and the type of hydroxyl substituent group can have an impact on the antioxidant activity of EPs and NEPs.

To further investigate NEPs in F&Veg wastes, it should be noted that degradation, incomplete hydrolysis induced during NEPs extraction, and the limited methods available for the identification and quantification of phenolic compounds can make this aspect of the study difficult. In such cases, the extracted NEPs should be analyzed using different analytical techniques to determine its chemical composition and its antioxidant properties. Depending on the results, NEPs can be further applied to food and pharmaceutical products.

Notably, NEPs have clear advantages in improving intestinal health. Compared to EPs, they retain a more intact structure in the intestine, maintain stable biological activity and have a good modulating effect on gastrointestinal digestion and colonic fermentation processes, but the difficulty of releasing NEPs has also become a challenge for research. Pretreatment of NEPs is recommended to make them more easily hydrolyzed and absorbed by the body. NEPs can ameliorate oxidative stress and impair intestinal barrier function by modulating immune function, enhancing antioxidant enzyme activity, balancing inflammatory factors, upregulating tight junction protein expression, and regulating intestinal flora, but there are complex interactions between these mechanisms that need further study. Therefore, the study of the effects of NEPs on oxidative stress-induced impaired intestinal barrier function and the linkage between different mechanisms of action will be the direction of future research, which is of great research significance for the development and utilization of NEPs.

The untapped potential of phenolic compounds in F&Veg waste provides a unique opportunity to strike a balance between environmental sustainability and economic growth. The full exploitation of phenolic compounds presents in F&Veg wastes, in conjunction with advanced natural extracting processes, holds the potential to significantly reduce the environmental footprint of human activities. Furthermore, the extraction of functional components from F&Veg waste has the potential to generate new value-added products for various sectors, e.g., food, pharmaceutical, and chemical industries. This, in turn, could contribute to the reduction of waste management and promote the development of a circular economy, where resources are used, recovered, and reused in a sustainable manner. 

## Figures and Tables

**Table 3:** Structural formula of several common anthocyanins.

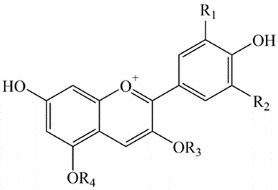		
**Anthocyanidin**	**R_1_**	**R_2_**
Pelargonidin	H	H
Delphinidin	OH	OH
Cyanidin	OH	H
Peonidin	OCH_3_	H
Petunidin	OCH_3_	OH
Malvidin	OCH_3_	OCH_3_

R1, R2, R3, R4 are all substituents on the benzene ring, R1, R2 represents H, OH or OCH_3_ and R3, R4 represents H or glycosyl substituents.

## Data Availability

Not applicable.

## Abbreviations

F&Vegs, fruit and vegetables; NEPs, non-extractable phenolic compounds; EPs, extractable phenolic compounds; NEPAs, non-extractable proanthocyanidins; EPAs, extractable proanthocyanidins; BHA, butylated hydroxyanisole; BHT, butylated hydroxytoluene; HPLC, high-performance liquid chromatography; PAs, proanthocyanidins; HTs, hydrolyzed tannins; HPPs, hydrolyzable phenolic compounds; mDP, mean average degree of polymerization, HSCCC, high-speed counter-current chromatography; HPTLC, high-performance thin-layer chromatography; NF-κB, nuclear factor-κB; NO, nitric oxide; UC, ulcerative colitis; TNF-α, tumor necrosis factor-α, IL-β, interleukin-β; IL-6, interleukin-6; IL-10, interleukin-10; MDA, Malondialdehyde; T-SOD, total superoxide dismutase; iNOS, inducible nitric oxide synthase.

## References

[1] Asgar M.D.A. (2013). Anti-Diabetic Potential of Phenolic Compounds: A Review. Int. J. Food Prop..

[2] Fresco P., Borges F., Diniz C., Marques M.P.M. (2006). New Insights on the Anticancer Properties of Dietary Polyphenols. Med. Res. Rev..

[3] Ferrazzano G.F., Amato I., Ingenito A., Zarrelli A., Pinto G., Pollio A. (2011). Plant Polyphenols and Their Anti-Cariogenic Properties: A Review. Molecules.

[4] Yahfoufi N., Alsadi N., Jambi M., Matar C. (2018). The Immunomodulatory and Anti-Inflammatory Role of Polyphenols. Nutrients.

[5] Scalbert A., Johnson I.T., Saltmarsh M. (2005). Polyphenols: Antioxidants and Beyond. Am. J. Clin. Nutr..

[6] Domínguez-Rodríguez G., Marina M.L., Plaza M. (2017). Strategies for the Extraction and Analysis of Non-Extractable Polyphenols from Plants. J. Chromatogr. A.

[7] Laganà P., Anastasi G., Marano F., Piccione S., Singla R.K., Dubey A.K., Delia S., Coniglio M.A., Facciolà A., Di Pietro A. (2019). Phenolic Substances in Foods: Health Effects as Anti-Inflammatory and Antimicrobial Agents. J. AOAC Int..

[8] Singla R.K., Dubey A.K., Garg A., Sharma R.K., Fiorino M., Ameen S.M., Haddad M.A., Al-Hiary M. (2019). Natural Polyphenols: Chemical Classification, Definition of Classes, Subcategories, and Structures. J. AOAC Int..

[9] Gil M.I., Tomás-Barberán F.A., Hess-Pierce B., Kader A.A. (2002). Antioxidant Capacities, Phenolic Compounds, Carotenoids, and Vitamin C Contents of Nectarine, Peach, and Plum Cultivars from California. J. Agric. Food Chem..

[10] Yan Z., Zhong Y., Duan Y., Chen Q., Li F. (2020). Antioxidant Mechanism of Tea Polyphenols and Its Impact on Health Benefits. Anim. Nutr..

[11] Ganesan K., Xu B. (2017). A Critical Review on Polyphenols and Health Benefits of Black Soybeans. Nutrients.

[12] Olszowy M. (2019). What Is Responsible for Antioxidant Properties of Polyphenolic Compounds from Plants ?. Plant Physiol. Biochem..

[13] de Lima Cherubim D.J., Buzanello Martins C.V., Oliveira Fariña L., da Silva de Lucca R.A. (2020). Polyphenols as Natural Antioxidants in Cosmetics Applications. J. Cosmet. Dermatol..

[14] Sagar N.A., Pareek S., Sharma S., Yahia E.M., Lobo M.G. (2018). Fruit and Vegetable Waste: Bioactive Compounds, Their Extraction, and Possible Utilization. Compr. Rev. Food Sci. Food Saf..

[15] Liu X., Le Bourvellec C., Renard C.M.G.C. (2020). Interactions between Cell Wall Polysaccharides and Polyphenols: Effect of Molecular Internal Structure. Compr. Rev. Food Sci. Food Saf..

[16] Arranz S., Saura-Calixto F., Shaha S., Kroon P.A. (2009). High Contents of Nonextractable Polyphenols in Fruits Suggest That Polyphenol Contents of Plant Foods Have Been Underestimated. J. Agric. Food Chem..

[17] Martins C.C., Rodrigues R.C., Mercali G.D., Rodrigues E. (2022). New Insights into Non-Extractable Phenolic Compounds Analysis. Food Res. Int..

[18] Pérez-Jiménez J., Saura-Calixto F. (2018). Fruit Peels as Sources of Non-Extractable Polyphenols or Macromolecular Antioxidants: Analysis and Nutritional Implications. Food Res. Int..

[19] Sharma P., Gaur V.K., Kim S.H., Pandey A. (2020). Microbial Strategies for Bio-Transforming Food Waste into Resources. Bioresour. Technol..

[20] Gulcin İ. (2020). Antioxidants and Antioxidant Methods: An Updated Overview. Arch. Toxicol..

[21] Kumar H., Bhardwaj K., Sharma R., Nepovimova E. (2020). Fruit and Vegetable Peels: Utilization of High Value. Molecules.

[22] Parfitt J., Barthel M., MacNaughton S. (2010). Food Waste within Food Supply Chains: Quantification and Potential for Change to 2050. Philos. Trans. R. Soc. B Biol. Sci..

[23] Liu X., Le Bourvellec C., Yu J., Zhao L., Wang K., Tao Y., Renard C.M.G.C., Hu Z. (2022). Trends and Challenges on Fruit and Vegetable Processing: Insights into Sustainable, Traceable, Precise, Healthy, Intelligent, Personalized and Local Innovative Food Products. Trends Food Sci. Technol..

[24] Hussain S., Jõudu I., Bhat R. (2020). Dietary Fiber from Underutilized Plant Resources-A Positive Approach for Valorization of Fruit and Vegetable Wastes. Sustain.

[25] Montenegro-Landívar M.F., Tapia-Quirós P., Vecino X., Reig M., Valderrama C., Granados M., Cortina J.L., Saurina J. (2021). Fruit and Vegetable Processing Wastes as Natural Sources of Antioxidant-Rich Extracts: Evaluation of Advanced Extraction Technologies by Surface Response Methodology. J. Environ. Chem. Eng..

[26] Singh B., Singh J.P., Kaur A., Singh N. (2020). Phenolic Composition, Antioxidant Potential and Health Benefits of Citrus Peel. Food Res. Int..

[27] Li S., Xiao J., Chen L., Hu C., Chen P., Xie B., Sun Z. (2012). Identification of A-Series Oligomeric Procyanidins from Pericarp of Litchi Chinensis by FT-ICR-MS and LC-MS. Food Chem..

[28] Domínguez-Rodríguez G., Marina M.L., Plaza M. (2022). In Vitro Assessment of the Bioavailability of Bioactive Non-Extractable Polyphenols Obtained by Pressurized Liquid Extraction Combined with Enzymatic-Assisted Extraction from Sweet Cherry (*Prunus avium* L.). Pomace. Food Chem..

[29] Plaza M., Dominguez-Rodriguez G., Sahelices C., Luisa Marina M. (2021). A Sustainable Approach for Extracting Non-Extractable Phenolic Compounds from Mangosteen Peel Using Ultrasound-Assisted Extraction and Natural Deep Eutectic Solvents. Appl. Sci..

[30] Sun S., Huang S., Shi Y., Shao Y., Qiu J., Sedjoah R.-C.A.-A., Yan Z., Ding L., Zou D., Xin Z. (2021). Extraction, Isolation, Characterization and Antimicrobial Activities of Non-Extractable Polyphenols from Pomegranate Peel. Food Chem..

[31] Cheng A., Chen X., Wang W., Gong Z., Liu L. (2013). Contents of Extractable and Non-Extractable Polyphenols in the Leaves of Blueberry. Czech J. Food Sci..

[32] Bouarab Chibane L., Degraeve P., Ferhout H., Bouajila J., Oulahal N. (2019). Plant Antimicrobial Polyphenols as Potential Natural Food Preservatives. J. Sci. Food Agric..

[33] Zhang Z., Li X., Sang S., McClements D.J., Chen L., Long J., Jiao A., Jin Z., Qiu C. (2022). Polyphenols as Plant-Based Nutraceuticals: Health Effects, Encapsulation, Nano-Delivery, and Application. Foods.

[34] Gonzales G.B., Raes K., Vanhoutte H., Coelus S., Smagghe G., Van Camp J. (2015). Liquid Chromatography–Mass Spectrometry Coupled with Multivariate Analysis for the Characterization and Discrimination of Extractable and Nonextractable Polyphenols and Glucosinolates from Red Cabbage and Brussels Sprout Waste Streams. J. Chromatogr. A.

[35] Pérez-Jiménez J., Díaz-Rubio M.E., Saura-Calixto F. (2013). Non-Extractable Polyphenols, a Major Dietary Antioxidant: Occurrence, Metabolic Fate and Health Effects. Nutr. Res. Rev..

[36] Ding Y., Morozova K., Scampicchio M., Ferrentino G. (2020). Non-Extractable Polyphenols from Food by-Products: Current Knowledge on Recovery, Characterisation, and Potential Applications. Processes.

[37] Ribas-Agustí A., Martín-Belloso O., Soliva-Fortuny R., Elez-Martínez P. (2018). Food Processing Strategies to Enhance Phenolic Compounds Bioaccessibility and Bioavailability in Plant-Based Foods. Crit. Rev. Food Sci. Nutr..

[38] Romano R., Aiello A., Meca G., De Luca L., Pizzolongo F., Masi P. (2021). Recovery of Bioactive Compounds from Walnut (*Juglans Regia* L.) Green Husk by Supercritical Carbon Dioxide Extraction. Int. J. Food Sci. Technol..

[39] Esparza-Martínez F.J., Miranda-López R., Mata-Sánchez S.M., Guzmán-Maldonado S.H. (2016). Extractable and Non-Extractable Phenolics and Antioxidant Capacity of Mandarin Waste Dried at Different Temperatures. Plant Foods Hum. Nutr..

[40] Vadez-Morales M., Gabriela Espinosa-Alonso L., Citlali Espinoza-Torres L., Delgado-Vargas F., Medina-Godoy S. (2014). Phenolic Content and Antioxidant and Antimutagenic Activities in Tomato Peel, Seeds, and Byproducts. J. Agric. Food Chem..

[41] Aires A., Carvalho R., Saavedra M.J. (2017). Reuse Potential of Vegetable Wastes (Broccoli, Green Bean and Tomato) for the Recovery of Antioxidant Phenolic Acids and Flavonoids. Int. J. Food Sci. Technol..

[42] Torres M.D., Dominguez H. (2020). Advances in Recovery Bioactive Compounds from Potato Wastes: Processing Technologies and Applications. Int. J. Food Sci. Technol..

[43] Calani L., Bresciani L., Rodolfi M., Del Rio D., Petruccelli R., Faraloni C., Ganino T. (2022). Characterization of the (Poly)Phenolic Fraction of Fig Peel: Comparison among Twelve Cultivars Harvested in Tuscany. Plants.

[44] Mauro R.P., Agnello M., Rizzo V., Graziani G., Fogliano V., Leonardi C., Giuffrida F. (2020). Recovery of Eggplant Field Waste as a Source of Phytochemicals. Sci. Hortic..

[45] Fernandes P.A.R., Le Bourvellec C., Renard C.M.G.C., Nunes F.M., Bastos R., Coelho E., Wessel D.F., Coimbra M.A., Cardoso S.M. (2019). Revisiting the Chemistry of Apple Pomace Polyphenols. Food Chem..

[46] Gunes R., Palabiyik I., Toker O.S., Konar N., Kurultay S. (2019). Incorporation of Defatted Apple Seeds in Chewing Gum System and Phloridzin Dissolution Kinetics. J. Food Eng..

[47] Liu X., Le Bourvellec C., Guyot S., Renard C.M.G.C. (2021). Reactivity of Flavanols: Their Fate in Physical Food Processing and Recent Advances in Their Analysis by Depolymerization. Compr. Rev. Food Sci. Food Saf..

[48] Dzah C.S., Duan Y., Zhang H., Serwah Boateng N.A., Ma H. (2020). Latest Developments in Polyphenol Recovery and Purification from Plant By-Products: A Review. Trends Food Sci. Technol..

[49] Ramirez-Bolanos S., Perez-Jimenez J., Diaz S., Robaina L. (2021). A Potential of Banana Flower and Pseudo-Stem as Novel Ingredients Rich in Phenolic Compounds. Int. J. Food Sci. Technol..

[50] Tow W.W., Premier R., Jing H., Ajlouni S. (2011). Antioxidant and Antiproliferation Effects of Extractable and Nonextractable Polyphenols Isolated from Apple Waste Using Different Extraction Methods. J. Food Sci..

[51] Tremocoldi M.A., Rosalen P.L., Franchin M., Massarioli A.P., Denny C., Daiuto É.R., Paschoal J.A.R., Melo P.S., de Alencar S.M. (2018). Exploration of Avocado By-Products as Natural Sources of Bioactive Compounds. PLoS One.

[52] Begum Y.A., Deka S.C. (2019). Chemical Profiling and Functional Properties of Dietary Fibre Rich Inner and Outer Bracts of Culinary Banana Flower. J. Food Sci. Technol..

[53] Reynoso-Camacho R., Sotelo-González A.M., Patiño-Ortiz P., Rocha-Guzmán N.E., Pérez-Ramírez I.F. (2021). Berry By-Products Obtained from a Decoction Process Are a Rich Source of Low- and High-Molecular Weight Extractable and Non-Extractable Polyphenols. Food Bioprod. Process..

[54] Struck S., Plaza M., Turner C., Rohm H. (2016). Berry Pomace—A Review of Processing and Chemical Analysis of Its Polyphenols. Int. J. Food Sci. Technol..

[55] Resende L.M., Franca A.S., Oliveira L.S. (2019). Buriti (Mauritia Flexuosa L. f.) Fruit by-Products Flours: Evaluation as Source of Dietary Fibers and Natural Antioxidants. Food Chem..

[56] Vella F.M., Cautela D., Laratta B. (2019). Characterization of Polyphenolic Compounds in Cantaloupe Melon By-Products. Foods.

[57] Fundo J.F., Miller F.A., Garcia E., Santos J.R., Silva C.L.M., Brandão T.R.S. (2018). Physicochemical Characteristics, Bioactive Compounds and Antioxidant Activity in Juice, Pulp, Peel and Seeds of Cantaloupe Melon. J. Food Meas. Charact..

[58] Martínez-Meza Y., Pérez-Jiménez J., Rocha-Guzmán N.E., Rodríguez-García M.E., Alonzo-Macías M., Reynoso-Camacho R. (2021). Modification on the Polyphenols and Dietary Fiber Content of Grape Pomace by Instant Controlled Pressure Drop. Food Chem..

[59] Martínez-Meza Y., Pérez-Jiménez J., Castaño-Tostado E., Pérez-Ramírez I.F., Alonzo-Macías M., Reynoso-Camacho R. (2022). Instant Controlled Pressure Drop as a Strategy To Modify Extractable and Non-Extractable Phenolic Compounds: A Study in Different Grape Pomace Materials. J. Agric. Food Chem..

[60] Reynoso-Camacho R., Rodriguez-Villanueva L.D., Sotelo-Gonzalez A.M., Ramos-Gomez M., Perez-Ramirez I.F. (2021). Citrus Decoction By-Product Represents a Rich Source of Carotenoid, Phytosterol, Extractable and Non-Extractable Polyphenols. Food Chem..

[61] Gülcü M., Uslu N., Özcan M.M., Gökmen F., Özcan M.M., Banjanin T., Gezgin S., Dursun N., Geçgel Ü., Ceylan D.A. (2019). The Investigation of Bioactive Compounds of Wine, Grape Juice and Boiled Grape Juice Wastes. J. Food Process. Preserv..

[62] Teng X., Zhang M., Mujumdar A.S., Wang H. (2022). Inhibition of Nitrite in Prepared Dish of *Brassica Chinensis* L. during Storage via Non-Extractable Phenols in Hawthorn Pomace: A Comparison of Different Extraction Methods. Food Chem..

[63] Lin S., Li Q., Yang B., Duan X., Zhang M., Shi J., Jiang Y. (2016). Transformation of Litchi Pericarp-Derived Condensed Tannin with Aspergillus Awamori. Int. J. Mol. Sci..

[64] Yang Z., Zhang L., Wu Y.-H., Li D.-P., Li W. (2022). Evaluation of Chemical Constituents of Litchi Pericarp Extracts and Its Antioxidant Activity in Mice. Foods.

[65] Di Donato P., Taurisano V., Tommonaro G., Pasquale V., Jiménez J.M.S., de Pascual-Teresa S., Poli A., Nicolaus B. (2018). Biological Properties of Polyphenols Extracts from Agro Industry’s Wastes. Waste Biomass Valorization.

[66] Multari S., Carlin S., Sicari V., Martens S. (2020). Differences in the Composition of Phenolic Compounds, Carotenoids, and Volatiles between Juice and Pomace of Four Citrus Fruits from Southern Italy. Eur. Food Res. Technol..

[67] Pfukwa T.M., Fawole O.A., Manley M., Gouws P.A., Opara U.L., Mapiye C. (2019). Food Preservative Capabilities of Grape (*Vitis Vinifera*) and Clementine Mandarin (*Citrus Reticulata*) By-Products Extracts in South Africa. Sustainability.

[68] Sáyago-Ayerdi S.G., Zamora-Gasga V.M., Venema K. (2019). Prebiotic Effect of Predigested Mango Peel on Gut Microbiota Assessed in a Dynamic in Vitro Model of the Human Colon (TIM-2). Food Res. Int..

[69] Suttirak W., Manurakchinakorn S. (2014). In Vitro Antioxidant Properties of Mangosteen Peel Extract. J. Food Sci. Technol..

[70] Freitas C.M.P., Sousa R.C.S., Dias M.M.S., Coimbra J.S.R. (2020). Extraction of Pectin from Passion Fruit Peel. Food Eng. Rev..

[71] Song Y., Wei X.-Q., Li M.-Y., Duan X.-W., Sun Y.-M., Yang R.-L., Su X.-D., Huang R.-M., Wang H. (2018). Nutritional Composition and Antioxidant Properties of the Fruits of a Chinese Wild Passiflora Foetida. Molecules.

[72] Kawakami S., Morinaga M., Tsukamoto-Sen S., Mori S., Matsui Y., Kawama T. (2022). Constituent Characteristics and Functional Properties of Passion Fruit Seed Extract. Life-Basel.

[73] Liu H., Jiang W., Cao J., Ma L. (2018). Evaluation of Antioxidant Properties of Extractable and Nonextractable Polyphenols in Peel and Flesh Tissue of Different Peach Varieties. J. Food Process. Preserv..

[74] de Camargo A.C., Regitano-d’Arce M.A.B., Rasera G.B., Canniatti-Brazaca S.G., do Prado-Silva L., Alvarenga V.O., Sant’Ana A.S., Shahidi F. (2017). Phenolic Acids and Flavonoids of Peanut By-Products: Antioxidant Capacity and Antimicrobial Effects. Food Chem..

[75] Cangussu L.B., Leao D.P., Oliveira L.S., Franca A.S. (2021). Profile of Bioactive Compounds in Pequi (Caryocar Brasilense Camb.) Peel Flours. Food Chem..

[76] Siqueira B. (2012). dos S.; Alves, L.D.; Vasconcelos, P.N.; Damiani, C.; Soares Júnior, M.S. Pectina extraída de casca de pequi e aplicação em geleia light de manga. Rev. Bras. Frutic..

[77] Savic I.M., Gajic I.M.S. (2022). Determination of Physico-Chemical and Functional Properties of Plum Seed Cakes for Estimation of Their Further Industrial Applications. Sustainability.

[78] Amaya-Cruz D.M., Pérez-Ramírez I.F., Delgado-García J., Mondragón-Jacobo C., Dector-Espinoza A., Reynoso-Camacho R. (2019). An Integral Profile of Bioactive Compounds and Functional Properties of Prickly Pear (*Opuntia Ficus Indica* L.) Peel with Different Tonalities. Food Chem..

[79] Özcan M.M., Uslu N., Kara H.H., Özcan M.M. (2022). Variations in Bioactive Properties, Phenolic Compounds and Fatty Acid Compositions of Different Parts of Prickly Pear (*Opuntia Ficus-Indica* Spp) Fruits. Erwerbs-Obstbau.

[80] Amaya-Cruz D., Perez-Ramirez I.F., Perez-Jimenez J., Nava G.M., Reynoso-Camacho R. (2019). Comparison of the Bioactive Potential of Roselle (*Hibiscus Sabdariffa* L.) Calyx and Its by-Product: Phenolic Characterization by UPLC-QTOF MSE and Their Anti-Obesity Effect in Vivo. Food Res. Int..

[81] Batley R.J., Johnson J.B., Mani J.S., Broszczak D.A., Naiker M. (2022). Finding Alternative Uses for Australian Rosella. Anim. Prod. Sci..

[82] Martínez-Inda B., Esparza I., Moler J.A., Jiménez-Moreno N., Ancín-Azpilicueta C. (2023). Valorization of Agri-Food Waste through the Extraction of Bioactive Molecules. Prediction of Their Sunscreen Action. J. Environ Manag..

[83] Kalogeropoulos N., Chiou A., Pyriochou V., Peristeraki A., Karathanos V.T. (2012). Bioactive Phytochemicals in Industrial Tomatoes and Their Processing Byproducts. Lwt-Food Sci. Technol..

[84] Domínguez-Rodríguez G., Ramón Vidal D., Martorell P., Plaza M., Marina M.L. (2022). Composition of Nonextractable Polyphenols from Sweet Cherry Pomace Determined by DART-Orbitrap-HRMS and Their In Vitro and In Vivo Potential Antioxidant, Antiaging, and Neuroprotective Activities. J. Agric. Food Chem..

[85] Nunes A.R., Gonçalves A.C., Pinto E., Amaro F., Flores-Félix J.D., Almeida A., Guedes de Pinho P., Falcão A., Alves G., Silva L.R. (2022). Mineral Content and Volatile Profiling of *Prunus Avium* L. (Sweet Cherry) By-Products from Fund&atilde;o Region (Portugal). Foods.

[86] Cangussu L.B., Fronza P., Franca A.S., Oliveira L.S. (2021). Chemical Characterization and Bioaccessibility Assessment of Bioactive Compounds from Umbu (*Spondias Tuberosa* A.) Fruit Peel and Pulp Flours. Foods.

[87] Yucetepe A., Altin G., Ozcelik B. (2021). A Novel Antioxidant Source: Evaluation of in Vitro Bioaccessibility, Antioxidant Activity and Polyphenol Profile of Phenolic Extract from Black Radish Peel Wastes (Raphanus Sativus L. Var. Niger) during Simulated Gastrointestinal Digestion. Int. J. Food Sci. Technol..

[88] Sadef Y., Javed T., Javed R., Mahmood A., Alwahibi M.S., Elshikh M.S., AbdelGawwa M.R., Alhaji J.H., Rasheed R.A. (2022). Nutritional Status, Antioxidant Activity and Total Phenolic Content of Different Fruits and Vegetables’ Peels. PLoS One.

[89] Lau W.K., Van Chuyen H., Vuong Q.V. (2018). Physical Properties, Carotenoids and Antioxidant Capacity of Carrot (*Daucus Carota* L.) Peel as Influenced by Different Drying Treatments. Int. J. Food Eng..

[90] Munir A., Sultana B., Bashir A., Ghaffar A., Munir B., Shar G.A., Nazir A., Iqbal M. (2018). Evaluation of Antioxidant Potential of Vegetables Waste. Polish J. Environ. Stud..

[91] Nasir G., Zaidi S., Tabassum N., Asfaq (2022). A Review on Nutritional Composition, Health Benefits and Potential Applications of by-Products from Pea Processing. Biomass Convers. Biorefinery.

[92] Boyano-Orozco L., Gallardo-Velázquez T., Meza-Márquez O.G., Osorio-Revilla G. (2020). Microencapsulation of Rambutan Peel Extract by Spray Drying. Foods.

[93] Habotta O.A., Dawood M.A.O., Kari Z.A., Tapingkae W., Van Doan H. (2022). Antioxidative and Immunostimulant Potential of Fruit Derived Biomolecules in Aquaculture. Fish Shellfish. Immunol..

[94] Chadorshabi S., Hallaj-Nezhadi S., Ghasempour Z. (2022). Red Onion Skin Active Ingredients, Extraction and Biological Properties for Functional Food Applications. Food Chem..

[95] Puganen A., Kallio H.P., Schaich K.M., Suomela J.-P., Yang B. (2018). Red/Green Currant and Sea Buckthorn Berry Press Residues as Potential Sources of Antioxidants for Food Use. J. Agric. Food Chem..

[96] Permal R., Chang W.L., Seale B., Hamid N., Kam R. (2020). Converting Industrial Organic Waste from the Cold-Pressed Avocado Oil Production Line into a Potential Food Preservative. Food Chem..

[97] De Santiago E., Juániz I., Cid C., De Peña M.-P. (2021). Extraction of (Poly)Phenolic Compounds of Cactus (*Opuntia ficus-indica* (L.) Mill.) Cladodes. Food Anal. Methods.

[98] Yan L., Zheng G. (2017). Comparing Profiles and Antioxidant Properties of Soluble and Insoluble Phenolics in Perilla Frutescens Seed Flour Extracts Obtained by Different Extraction/Hydrolysis Methods. Int. J. Food Sci. Technol..

[99] Marmol I., Quero J., Ibarz R., Ferreira-Santos P., Teixeira J.A., Rocha C.M.R., Perez-Fernandez M., Garcia-Juiz S., Osada J., Martin-Belloso O. (2021). Valorization of Agro-Food by-Products and Their Potential Therapeutic Applications. Food Bioprod. Process..

[100] Tang Y.-Y., He X.-M., Sun J., Li C.-B., Li L., Sheng J.-F., Xin M., Li Z.-C., Zheng F.-J., Liu G.-M. (2019). Polyphenols and Alkaloids in Byproducts of Longan Fruits (*Dimocarpus Longan* Lour.) and Their Bioactivities. Molecules.

[101] Cheng A., Yan H., Han C., Chen X., Wang W., Xie C., Qu J., Gong Z., Shi X. (2014). Acid and Alkaline Hydrolysis Extraction of Non-Extractabke Polyphenols in Blueberries Optimisation by Response Surface Methodology. Czech J. Food Sci..

[102] Wang L., Wu Y., Liu Y., Wu Z. (2017). Complex Enzyme-Assisted Extraction Releases Antioxidative Phenolic Compositions from Guava Leaves. Molecules.

[103] Domínguez-Rodríguez G., Plaza M., Marina M.L. (2021). High-Performance Thin-Layer Chromatography and Direct Analysis in Real Time-High Resolution Mass Spectrometry of Non-Extractable Polyphenols from Tropical Fruit Peels. Food Res. Int..

[104] Anokwuru C., Sigidi M., Boukandou M., Tshisikhawe P., Traore A., Potgieter N. (2018). Antioxidant Activity and Spectroscopic Characteristics of Extractable and Non-Extractable Phenolics from Terminalia Sericea Burch. Ex DC. Molecules.

[105] Gulsunoglu Z., Karbancioglu-Guler F., Raes K., Kilic-Akyilmaz M. (2019). Soluble and Insoluble-Bound Phenolics and Antioxidant Activity of Various Industrial Plant Wastes. Int. J. Food Prop..

[106] Durazzo A. (2017). Study Approach of Antioxidant Properties in Foods: Update and Considerations. Foods.

[107] Rojas-Garcia A., Rodriguez L., Cadiz-Gurrea M.d.l.L., Garcia-Villegas A., Fuentes E., Villegas-Aguilar M.d.C., Palomo I., Arraez-Roman D., Segura-Carretero A. (2022). Determination of the Bioactive Effect of Custard Apple By-Products by in Vitro Assays. Int. J. Mol. Sci..

[108] Mrabet A., Jimenez-Araujo A., Fernandez-Prior A., Bermudez-Oria A., Fernandez-Bolanos J., Sindic M., Rodriguez-Gutierrez G. (2022). Date Seed: Rich Source of Antioxidant Phenolics Obtained by Hydrothermal Treatments. Antioxidants.

[109] Rodríguez R., Jaramillo S., Rodríguez G., Espejo J.A., Guillén R., Fernández-Bolaños J., Heredia A., Jiménez A. (2005). Antioxidant Activity of Ethanolic Extracts from Several Asparagus Cultivars. J. Agric. Food Chem..

[110] Castaldo L., Izzo L., De Pascale S., Narvaez A., Rodriguez-Carrasco Y., Ritieni A. (2021). Chemical Composition, In Vitro Bioaccessibility and Antioxidant Activity of Polyphenolic Compounds from Nutraceutical Fennel Waste Extract. Molecules.

[111] Shimizu M. (2017). Multifunctions of Dietary Polyphenols in the Regulation of Intestinal Inflammation. J. Food Drug Anal..

[112] Li W., Yang H., Zhao Q., Wang X., Zhang J., Zhao X. (2019). Polyphenol-Rich Loquat Fruit Extract Prevents Fructose-Induced Nonalcoholic Fatty Liver Disease by Modulating Glycometabolism, Lipometabolism, Oxidative Stress, Inflammation, Intestinal Barrier, and Gut Microbiota in Mice. J. Agric. Food Chem..

[113] Bischoff S.C., Barbara G., Buurman W., Ockhuizen T., Schulzke J.-D., Serino M., Tilg H., Watson A., Wells J.M. (2014). Intestinal Permeability–A New Target for Disease Prevention and Therapy. BMC Gastroenterol..

[114] Yu L.C.-H., Wang J.-T., Wei S.-C., Ni Y.-H. (2012). Host-Microbial Interactions and Regulation of Intestinal Epithelial Barrier Function: From Physiology to Pathology. World J. Gastrointest. Pathophysiol..

[115] Huang Z., Yang W., Wang X., Guo F., Cheng Y., Cao L., Zhu W., Sun Y., Xiong H. (2022). Industrially Produced Rice Protein Ameliorates Dextran Sulfate Sodium-Induced Colitis via Protecting the Intestinal Barrier, Mitigating Oxidative Stress, and Regulating Gut Microbiota. J. Agric. Food Chem..

[116] Qiao Y., Sun J., Ding Y., Le G., Shi Y. (2013). Alterations of the Gut Microbiota in High-Fat Diet Mice Is Strongly Linked to Oxidative Stress. Appl. Microbiol. Biotechnol..

[117] Sholl J., Mailing L.J., Wood T.R. (2021). Reframing Nutritional Microbiota Studies to Reflect an Inherent Metabolic Flexibility of the Human Gut: A Narrative Review Focusing on High-Fat Diets. mBio.

[118] Rocchetti G., Gregorio R.P., Lorenzo J.M., Barba F.J., Oliveira P.G., Prieto M.A., Simal-Gandara J., Mosele J.I., Motilva M., Tomas M. (2022). Functional Implications of Bound Phenolic Compounds and Phenolics–Food Interaction: A Review. Compr. Rev. Food Sci. Food Saf..

[119] Williamson G., Clifford M.N. (2017). Role of the Small Intestine, Colon and Microbiota in Determining the Metabolic Fate of Polyphenols. Biochem. Pharmacol..

[120] Ordoñez-Díaz J.L., Moreno-Ortega A., Roldán-Guerra F.J., Ortíz-Somovilla V., Moreno-Rojas J.M., Pereira-Caro G. (2020). In Vitro Gastrointestinal Digestion and Colonic Catabolism of Mango (*Mangifera indica* L.) Pulp Polyphenols. Foods.

[121] Gong E.S., Gao N., Li T., Chen H., Wang Y., Si X., Tian J., Shu C., Luo S., Zhang J. (2019). Effect of in Vitro Digestion on Phytochemical Profiles and Cellular Antioxidant Activity of Whole Grains. J. Agric. Food Chem..

[122] Maurer L.H., Cazarin C.B.B., Quatrin A., Minuzzi N.M., Nichelle S.M., Lamas C.d.A., Cagnon V.H.A., Morari J., Velloso L.A., Júnior M.R.M. (2020). Grape Peel Powder Attenuates the Inflammatory and Oxidative Response of Experimental Colitis in Rats by Modulating the NF-ΚB Pathway and Activity of Antioxidant Enzymes. Nutr. Res..

[123] Huang B., Wang L., Liu M., Wu X., Lu Q., Liu R. (2022). The Underlying Mechanism of A-type Procyanidins from Peanut Skin on DSS-induced Ulcerative Colitis Mice by Regulating Gut Microbiota and Metabolism. J. Food Biochem..

[124] Durazzo A., Caiazzo E., Lucarini M., Cicala C., Izzo A.A., Novellino E., Santini A. (2019). Polyphenols: A Concise Overview on the Chemistry, Occurrence, and Human Health. Phyther. Res..

[125] Yao Y., Ren G. (2011). LWT—Food Science and Technology Effect of Thermal Treatment on Phenolic Composition and Antioxidant Activities of Two Celery Cultivars. LWT Food Sci. Technol..

[126] Cosme F., Pinto T., Vilela A. (2018). Phenolic Compounds and Antioxidant Activity in Grape Juices: A Chemical and Sensory View. Beverages.

[127] Šamec D., Karalija E., Šola I., Vujčić Bok V., Salopek-Sondi B. (2021). The Role of Polyphenols in Abiotic Stress Response: The Influence of Molecular Structure. Plants.

[128] Rodríguez-Bonilla P., Gandía-Herrero F., Matencio A., García-Carmona F., López-Nicolás J.M. (2017). Comparative Study of the Antioxidant Capacity of Four Stilbenes Using ORAC, ABTS+, and FRAP Techniques. Food Anal. Methods.

[129] Acids P. (1996). Structure-Antioxidant Activity Relationships of Flavonoids and Phenolic Acids. Free Radic. Biol. Med..

[130] Hirano R., Sasamoto W., Matsumoto A., Itakura H., Igarashi O., Kondo K. (2001). Antioxidant Ability of Various Flavonoids against DPPH Radicals and LDL Oxidation. J. Nutr. Sci. Vitaminol..

[131] Stanislaw B., Oleszek W. (2001). Antioxidant and Antiradical Activities of Flavonoids. J. Agric. Food Chem..

[132] Heim K.E., Tagliaferro A.R., Bobilya D.J. (2002). Flavonoid Antioxidants: Chemistry, Metabolism and Structure-Activity Relationships. J. Nutr. Biochem..

[133] van Acker S.A.B.E., de Groot M.J., van den Berg D.-J., Tromp M.N.J.L., Donné-Op den Kelder G., van der Vijgh W.J.F., Bast A. (1996). A Quantum Chemical Explanation of the Antioxidant Activity of Flavonoids. Chem. Res. Toxicol..

[134] Deng D., Zhang J., Cooney J.M., Skinner M.A., Adaim A., Jensen D.J., Stevenson D.E. (2006). Methylated Polyphenols Are Poor “Chemical” Antioxidants but Can Still Effectively Protect Cells from Hydrogen Peroxide-Induced Cytotoxicity. FEBS Lett..

[135] Su Y.L., Xu J.Z., Ng C.H., Leung L.K., Huang Y., Chen Z.Y. (2004). Antioxidant Activity of Tea Theaflavins and Methylated Catechins in Canola Oil. JAOCS, J. Am. Oil Chem. Soc..

[136] Dueñas M., González-Manzano S., González-Paramás A., Santos-Buelga C. (2010). Antioxidant Evaluation of O-Methylated Metabolites of Catechin, Epicatechin and Quercetin. J. Pharm. Biomed. Anal..

[137] Woodman O.L., Meeker W.F., Boujaoude M. (2005). Vasorelaxant and Antioxidant Activity of Flavonols and Flavones: Structure—Activity Relationships. J. Cardiovasc. Pharmacol..

[138] Spiegel M., Kapusta K., Kołodziejczyk W., Saloni J., Zbikowska B., Hill G.A., Sroka Z. (2020). Antioxidant Activity of Selected Phenolic Acids–Ferric Reducing Antioxidant Power Assay and QSAR Analysis of the Structural Features. Molecules.

[139] Plumb G.W., Price K.R., Williamson G. (1999). Antioxidant Properties of Flavonol Glycosides from Tea. Redox Rep..

[140] Van Acker S.A.B.E., Van Den Berg D.J., Tromp M.N.J.L., Griffioen D.H., Van Bennekom W.P., Van Der Vijgh W.J.F., Bast A. (1996). Structural Aspects of Antioxidant Activity of Flavonoids. Free Radic. Biol. Med..

[141] Fukumoto L.R., Mazza G. (2000). Assessing Antioxidant and Prooxidant Activities of Phenolic Compounds. J. Agric. Food Chem..

[142] Kähkönen M.P., Heinonen M. (2003). Antioxidant Activity of Anthocyanins and Their Aglycons. J. Agric. Food Chem..

[143] da Silva C.M.G., Contesini F.J., Sawaya A.C.H.F., Cabral E.C., da Silva Cunha I.B., Eberlin M.N., de Oliveira Carvalho P. (2013). Enhancement of the Antioxidant Activity of Orange and Lime Juices by Flavonoid Enzymatic De-Glycosylation. Food Res. Int..

